# Synaptic connectome of a neurosecretory network in the *Drosophila* brain

**DOI:** 10.1101/2024.08.28.609616

**Published:** 2024-08-29

**Authors:** Theresa H. McKim, Jayati Gera, Ariana J. Gayban, Nils Reinhard, Giulia Manoli, Selina Hilpert, Charlotte Helfrich-Förster, Meet Zandawala

**Affiliations:** 1 Integrative Neuroscience Program, University of Nevada Reno, Reno, 89557, NV, USA; 2 Department of Biology, University of Nevada Reno, Reno, 89557, NV, USA; 3 Neurobiology and Genetics, Theodor-Boveri Institute, Biocenter, University of Würzburg, 97074 Würzburg, Germany; 4 Department of Biochemistry and Molecular Biology, University of Nevada Reno, Reno, 89557, NV, USA

**Keywords:** Connectomics, endocrine signaling, neuropeptides, GPCR, sensory input, descending neurons, taste

## Abstract

Hormones mediate inter-organ signaling which is crucial in orchestrating diverse behaviors and physiological processes including sleep and activity, feeding, growth, metabolism and reproduction. The pars intercerebralis and pars lateralis in insects represent major hubs which contain neurosecretory cells (NSC) that produce various hormones. To obtain insight into how hormonal signaling is regulated, we have characterized the synaptic connectome of NSC in the adult *Drosophila* brain. Identification of neurons providing inputs to multiple NSC subtypes implicates diuretic hormone 44-expressing NSC as a major coordinator of physiology and behavior. Surprisingly, despite most NSC having dendrites in the subesophageal zone (primary taste processing center), gustatory inputs to NSC are largely indirect. We also deciphered pathways via which diverse olfactory inputs are relayed to NSC. Further, our analyses revealed substantial inputs from descending neurons to NSC, suggesting that descending neurons regulate both endocrine and motor output to synchronize physiological changes with appropriate behaviors. In contrast to NSC inputs, synaptic output from NSC is sparse and mostly mediated by corazonin NSC. Therefore, we additionally determine putative paracrine interconnectivity between NSC subtypes and hormonal pathways from NSC to peripheral tissues by analyzing single-cell transcriptomic datasets. Our comprehensive characterization of the *Drosophila* neurosecretory network connectome provides a platform to understand complex hormonal networks and how they orchestrate animal behaviors and physiology.

## Introduction

The endocrine or hormonal systems in animals play a pivotal role in regulating development and a multitude of physiological processes including growth, metabolism, and reproduction (Nässel and Zandawala, 2019). In addition, hormones can target neuronal circuits to modulate diverse behaviors ranging from feeding and locomotion to courtship and aggression (Kim et al., 2017, Schoofs et al., 2017, Nässel and Zandawala, 2022). Hormones also enable organisms to adapt to changing external environments and internal states by permitting communication between the nervous system and peripheral tissues. This inter-organ signaling is crucial in orchestrating the functions of different tissues to attain homeostasis (Droujinine and Perrimon, 2016). Given its importance, it is not surprising that disrupted endocrine signaling can result in several disorders including obesity, diabetes, hypertension, infertility and growth defects amongst others (Golden et al., 2009). Understanding the regulation of endocrine signaling can thus provide insights into the prevention or treatment of endocrine-related disorders.

Although hormones can be produced by several tissues, the nervous system represents a major source of hormones. In vertebrates, the hypothalamus and pituitary contain neurosecretory cells (NSC) that are the source of several neuropeptides/hormones. These hormones, the tissues producing them, and their target tissues are categorized into different axes which regulate distinct functions. For example, the hypothalamic-pituitary-adrenal (HPA) axis utilizes corticotropin-releasing hormone (CRH), adrenocorticotropic hormone, and cortisol to primarily regulate the stress response (Herman et al., 2016). The hypothalamic-pituitary-thyroid (HPT) axis controls growth and metabolism whereas the hypothalamic-pituitary-gonad (HPG) axis regulates reproductive processes, both of which utilize several different hormones (Brent, 2012, Plant, 2015). Interestingly, there is also interaction between these systems. For instance, stress-regulating CRH can act on the HPG axis to reduce the production of sex hormone and suppress gonadal function (Ferin, 1999, Maeda and Tsukamura, 2006). Hence, these axes are interconnected, which underscores the regulatory complexity of the vertebrate endocrine system.

**Figure 1:**
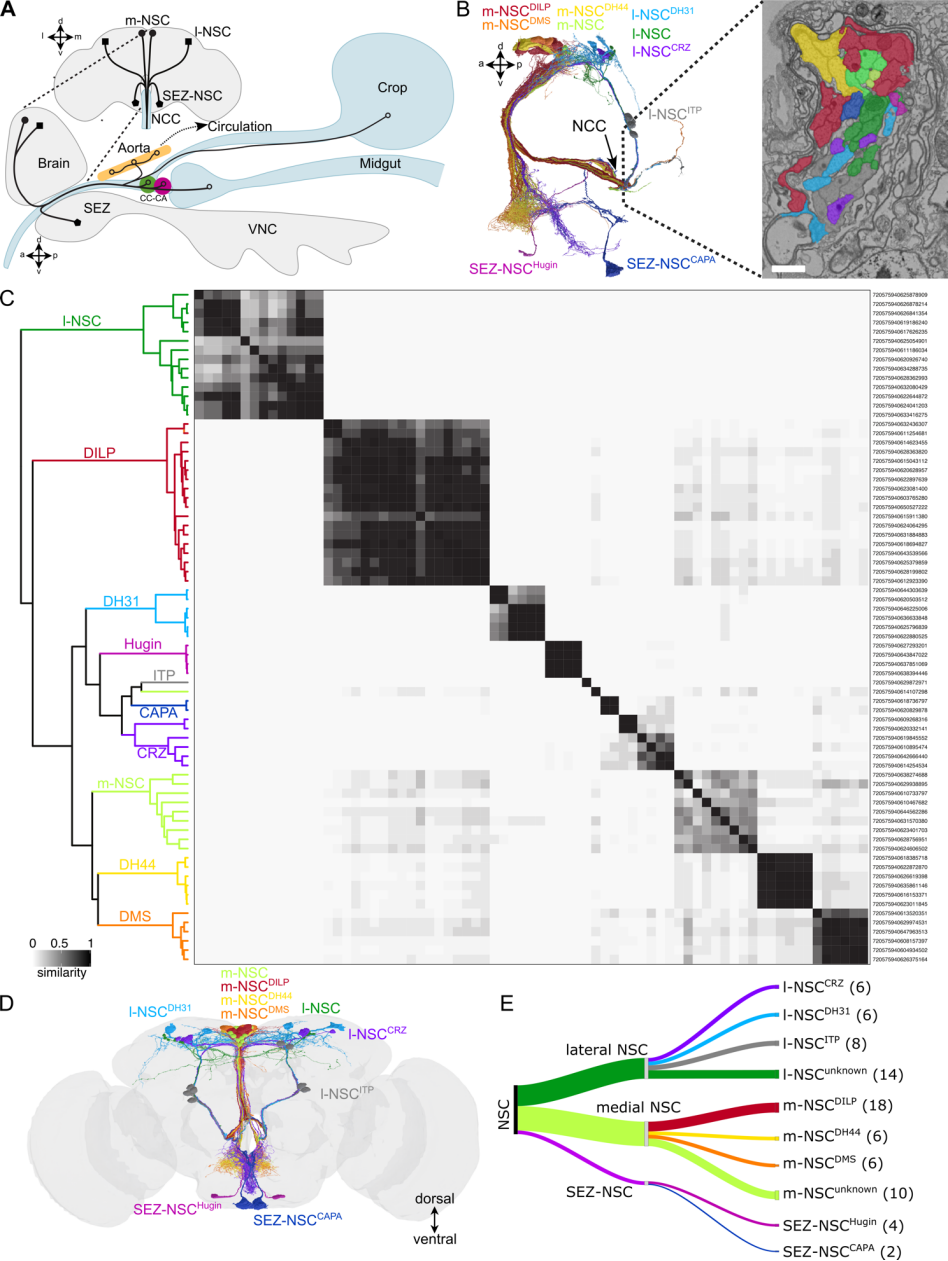
Identification of NSC in the *Drosophila* brain. **(A)** Schematic drawing of the different types of NSC and their projections to different release sites within the fly. Based on (Nässel et al., 2013). **(B)** All NSC projections exit the brain via the nervi corpora cardiaca (NCC). Electron micrograph showcasing a cross section of the NCC. Scale bar = 750nm. **(C)** Cosine similarity matrix of all NSC based on their total inputs. The darker the color, the higher the similarity between neurons. Neurons within the clades are colored based on the schematic in (D). **(D)** Reconstructions of the 80 NSC within the adult brain connectome. **(E)** Classification of NSC based on their location and neuropeptide expression. Refer to Table 1 for further details. Abbreviations: SEZ, subesophageal zone; CC, corpus cardiacum; CA, corpus allatum.

In contrast to vertebrates, the *Drosophila* brain contains a small number of NSC in the pars intercerebralis, pars lateralis, and subesophageal zone (SEZ). These NSC project their axons towards the corpora cardiaca (CC) and corpora allata (CA), a set of endocrine glands closely associated with the aorta and anterior gut (Figure 1A). Despite the large evolutionary timescale separating vertebrates and insects, there are similarities between their neuroendocrine systems (Nässel and Zandawala, 2020). These systems share significant similarities in structure, signaling pathways, and cell fate determinants during development (Hartenstein, 2006). Hence, the pars intercerebralis and CC are analogs of the vertebrate hypothalamus and pituitary, respectively. Strikingly, there is also conservation in some of the neuropeptides utilized by these systems. For instance, CRH is homologous to diuretic hormone 44 (DH44), and both hormones regulate stress responses (Furuya et al., 1995, Zandawala et al., 2018a, Nässel and Zandawala, 2019). Similarly, homologs of other hypothalamic neuropeptides such as prolactin-releasing peptide, neuromedin U, and gonadotropin-releasing hormone that regulate hormone release are also expressed in the *Drosophila* neuroendocrine system (Melcher et al., 2006, Zandawala et al., 2018b, Yanez-Guerra et al., 2020). Therefore, *Drosophila* with its smaller neuroendocrine system is an attractive model to unravel evolutionary conserved pathways which regulate hormonal signaling. While previous studies have characterized the connectomes of neuroendocrine centers in the larvae of *Drosophila* and *Platynereis dumerilii* (Williams et al., 2017, Huckesfeld et al., 2021), the neuroendocrine connectome of an adult animal is lacking. Given the expansion and transformation of the nervous system during metamorphosis, it remains to be seen which input pathways to NSC are conserved across animal development. Importantly, there are stark differences in physiology and behavior of larval and adult *Drosophila*. Therefore, the larval neuroendocrine system, which is mainly concerned with growth and development, is not entirely suitable to understand adult physiology and behavior.

To address this gap, we leveraged connectomics to characterize the first synaptic connectome of an adult neurosecretory network in an invertebrate. We deciphered all the major neuronal inputs to *Drosophila* NSC and focused on direct and indirect sensory input pathways to NSC. We also utilized single-cell transcriptomic analyses to explore potential paracrine interconnectivity between NSC subtypes, as well as endocrine inter-organ pathways. Our analyses shed light on the broader principles governing hormonal regulation and their impact on organismal physiology and behavior.

## Results

### Identification of cells comprising the neuroendocrine network in the FlyWire connectome

To characterize the synaptic connectome of the adult *Drosophila* neuroendocrine network, we first identified all endocrine or NSC in the brain which are a major source of circulating hormones. These endocrine cells can be broadly classified into lateral, medial, and subesophageal zone NSC (l-NSC, m-NSC, and SEZ-NSC, respectively) based on their location in the brain. Their axons exit the brain via a pair of nerves (nervii corpora cardiaca, NCC), and depending on the cell type, innervate the CC, CA, hypocerebral ganglion, crop, aorta, or the anterior midgut (Figure 1A). Their axon terminals form neurohemal sites through which hormones are released into the circulation or locally on peripheral targets such as the crop. Collectively, the NSC in the brain form a major, yet distributed, neuroendocrine network that is functionally analogous to the hypothalamus (Nässel and Zandawala, 2020). We identified all brain NSC in the FlyWire connectome by isolating the nerve bundle containing their axons (Figure 1B). In total, we independently identified 80 brain NSC, in agreement with our companion studies (Dorkenwald et al., 2023, Schlegel et al., 2023). We propose and utilize a systematic nomenclature for all brain NSC based on their location and neuropeptide identity (Table 1).

### Classification of NSC based on their morphology, neuropeptide identity, and synaptic connectivity

Interestingly, the number of NSC identified in the adult brain connectome is larger than the number of NSC characterized in the neural connectome of the first instar larvae (Huckesfeld et al., 2021) (Table 1). In larvae, two groups of m-NSC express myosuppressin (m-NSC^DMS^) and diuretic hormone 44 (m-NSC^DH44^). A third group of m-NSC express insulin-like peptides 2, 3, and 5 (m-NSC^DILP^), and are commonly referred to as insulin-producing cells. In addition, there are five groups of l-NSC which express ion-transport peptide (l-NSC^ITP^), corazonin (l-NSC^CRZ^), diuretic hormone 31 (l-NSC^DH31^), prothoracicotropic hormone and eclosion hormone. The latter two populations undergo apoptosis soon after adult eclosion and are thus not found in mature adults (Nässel and Zandawala, 2019). Lastly, the SEZ-NSC include two groups which express CAPA (SEZ-NSC^CAPA^) and Hugin (SEZ-NSC^Hugin^) neuropeptides. While 14 l-NSC^CRZ^ are present in adults, it is not clear if all of these are bona fide NSC and release CRZ into the circulation. Since the number of neurons comprising the remaining NSC subtypes is thought to remain constant across development, the identity of additional NSC found in adults needed to be clarified.

With this aim in mind, we sought to classify the adult NSC into different subtypes based on their neuropeptide identity. All SEZ-NSC and some l-NSC subtypes can easily be identified based on their morphology and location (Figure 1A). However, this approach is not feasible for m-NSC since they are clustered together in the superior medial protocerebrum and appear similar based on gross morphology.

**Figure 2:**
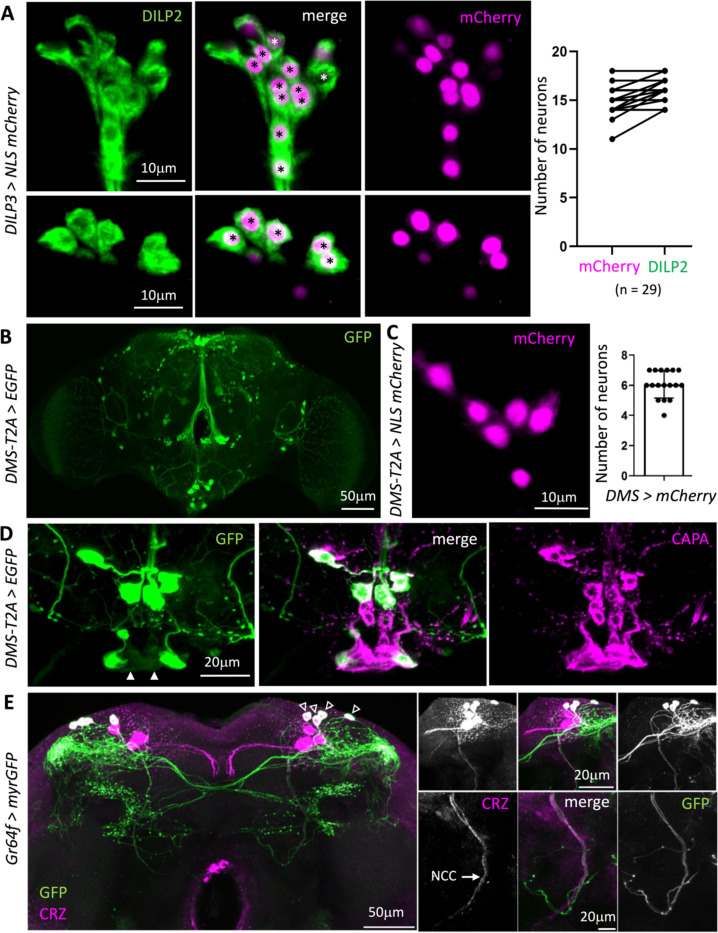
Quantification of NSC. **(A)** On average, there are 16 m-NSC^DILP^ as labelled by *DILP3-Gal4* and DILP2 antibody. Note that some preparations contain 18 m-NSC^DILP^, in agreement with the number determined based on the connectome. **(B)** *MS-T2A-Gal4* drives expression in several neuronal populations across the brain including **(C)** six m-NSC in pars intercerebralis and **(D)** the pair of SEZ-NSC^CAPA^ (filled arrowheads). **(E)** CRZ is expressed in 7 pairs of neurons in adult flies, 4 of which co-express *Gr64a* (empty arrowheads). These smaller *Gr64a*-expressing CRZ neurons form dense arborizations in the lateral horn. They project contralaterally but do not send projections via the nervii corpora cardiaca (NCC) and are thus not considered neurosecretory.

Therefore, we asked whether clustering NSC based on cosine similarity between their synaptic inputs can help distinguish and identify different NSC populations. We have recently utilized a similar approach to successfully classify neurons of the circadian clock (Reinhard et al., 2024). As expected, SEZ-NSC^Hugin^, SEZ-NSC^CAPA^, and l-NSC^DH31^ form three separate clusters (Figure 1C). Most l-NSC^ITP^ do not have any input synapses in this dataset and were thus excluded from the analysis. However, the 8 l-NSC^ITP^ are easily recognizable based on their unique morphology (Figure 1D-E). Notably, our clustering analysis resulted in two clades of m-NSC each comprising 6 neurons (Figure 1C). These clusters likely represent m-NSC^DMS^ (addressed below) and m-NSC^DH44^. We also obtained two additional clusters of m-NSC comprised of 18 and 10 neurons, with the latter having low similarity between the neurons forming that cluster. We suspected that the cluster comprised of 18 m-NSC represents m-NSC^DILP^ as we expected at least 14 m-NSC^DILP^ in the connectome. To clarify the number of m-NSC^DILP^ in adults, we quantified the number of cells labelled by DILP2 antibody in a *DILP3 > NLS mCherry* background (Figure 2A). On average, we detected 16 m-NSC^DILP^, with some preparations containing 18 neurons and most of them containing greater than 13 neurons. Hence, the largest m-NSC cluster represents m-NSC^DILP^. Similarly, we also quantified the number of m-NSC^DMS^ since we did not retrieve any clusters with 4 neurons as was anticipated for m-NSC^DMS^. *DMS-T2A-Gal4* drives GFP expression in 6 pars intercerebralis neurons on average which project via the NCC (Figure 2B-C). Surprisingly, we also detected GFP expression in SEZ-NSC^CAPA^ (Figure 2B and 2D). Thus, there are additional m-NSC^DILP^ and m-NSC^DMS^ neurons in adults compared to larvae, and circulating/hormonal DMS can originate from two NSC subtypes.

Intriguingly, one of the m-NSC clusters comprising 6 neurons represents m-NSC^DMS^ while the other represents m-NSC^DH44^. Since our clustering was based on input synapses, we reasoned that the differences in their pre-synaptic partners could enable us to identify these clusters. To explore this possibility, we performed retrograde trans-synaptic labelling of m-NSC^DH44^ neurons with retro-Tango (Sorkac et al., 2023) using a highly-specific *DH44-Gal4* (Figure 1 Supplement 1A). The majority of the input to m-NSC^DH44^ originates from neurons in the SEZ. Since no specific Gal4 drivers for m-NSC^DMS^ are currently available, we could not clearly map their presynaptic partners using retro-Tango. We next compared the retro-Tango output with the *in silico* retrograde tracing of 6 neurons belonging to each of the two m-NSC clusters (Figure 1 Supplement 1B-C). Surprisingly, both sets of m-NSC receive a majority of their inputs from neurons in the SEZ which have similar location and morphology. However, one set of m-NSC receives inputs from a group of central neurons (Figure 1 Supplement 1C) that are not visible with *DH44 > retro-Tango*. We therefore refer to this cluster as m-NSC^DMS^ (Figure 1 Supplement 1C) and the other cluster as m-NSC^DH44^ (Figure 1 Supplement 1B). To obtain additional support for our classification, we next focused on their ultrastructural features, specifically their dense core vesicles (DCV). A recent study has shown that vesicles for different neurotransmitters can exhibit slight but significant differences that are visible in electron micrographs (Eckstein et al., 2024). We thus asked whether visual or morphological differences in DCV are also observed for cells expressing DH44 and DMS. We addressed this by first identifying a pair of descending neurons in the connectome (Figure 1 Supplement 1D) which have previously been shown to express DMS (Carlsson et al., 2010). DCV within these neurons are clearly visible in the soma (Figure 1 Supplement 1E). These DCV appear to be heterogenous as they are of different sizes and contrast, suggesting presence of another neuromodulator in addition to DMS. Nonetheless, the higher contrast of the some DCV in the DMS descending neuron is more similar to that of DCV in m-NSC^DMS^ (Figure 1 Supplement 1G) compared to m-NSC^DH44^ (Figure 1 Supplement 1F), lending further support to their classification.

Lastly, we could only reliably identify 6 out of the expected 14 l-NSC^CRZ^ (Figure 1C and Table 1). Our inability to identify the remaining 8 CRZ neurons prompted us to examine if these adult-specific CRZ neurons are indeed neurosecretory. Previous work has shown that CRZ neurons express *Gr64a* and *Gr43a* gustatory receptors (Miyamoto and Amrein, 2014, Fujii et al., 2015). Using *Gr64a-Gal4* to label the adult-specific CRZ neurons (Fujii et al., 2015), we showed that there are only 6 adult l-NSC^CRZ^ (Figure 2E). These adult-specific CRZ neurons do not project via the NCC and are thus not neurosecretory. Hence, our clustering analysis accounts for all the NSC that persist into adulthood. Further, we uncovered 10 additional putative m-NSC and 14 additional putative l-NSC in the adult brain (Figure 1C-E). These neurons, especially l-NSC^unknown^, have relatively fewer DCV than neurons such as l-NSC^ITP^ (data not shown). Hence, the type (neuropeptide, biogenic amine, or fast-acting neurotransmitter) and the identity of the signaling molecules within these neurons remain unknown. Taken together, some NSC types have expanded in number in adults, along with an additional population of l-NSC and m-NSC (Figure 1D-E).

### Heterogeneity within the NSC subtypes

Having classified NSC into 10 subtypes, we next compared their morphological characteristics including their cable length, surface area, cell size, and nuclei volume (Figure 1 Supplement 2). SEZ-NSC^CAPA^ are about twice as large compared to other NSC types (Figure 1 Supplement 2C). The function of pyrokinin neuropeptide produced by these cells is still unknown (Wegener et al., 2006). But given their location, large size, and presumed large release capacity, we speculate that SEZ-NSC^CAPA^ participate in global modulation of post-feeding physiology. Additionally, we performed principal component analysis of the four morphological features, which revealed that the NSC of a given subtype generally cluster together. However, we also observed high variability within l-NSC^CRZ^, l-NSC^DH31^, m-NSC^DH44^, and m-NSC^DILP^ populations, indicating that they are comprised of morphologically heterogenous subpopulations. Clustering based on synaptic connectivity also supports this heterogeneity as we observed multiple subclades for these NSC types (Figure 1C). For instance, the 6 l-NSC^CRZ^ cluster into two separate subclades as they represent a heterogeneous population both anatomically and functionally (Oh et al., 2019, Zandawala et al., 2021). Thus, some of the 10 NSC subtypes classified here are heterogenous.

### Deciphering input pathways to NSC

**Figure 3:**
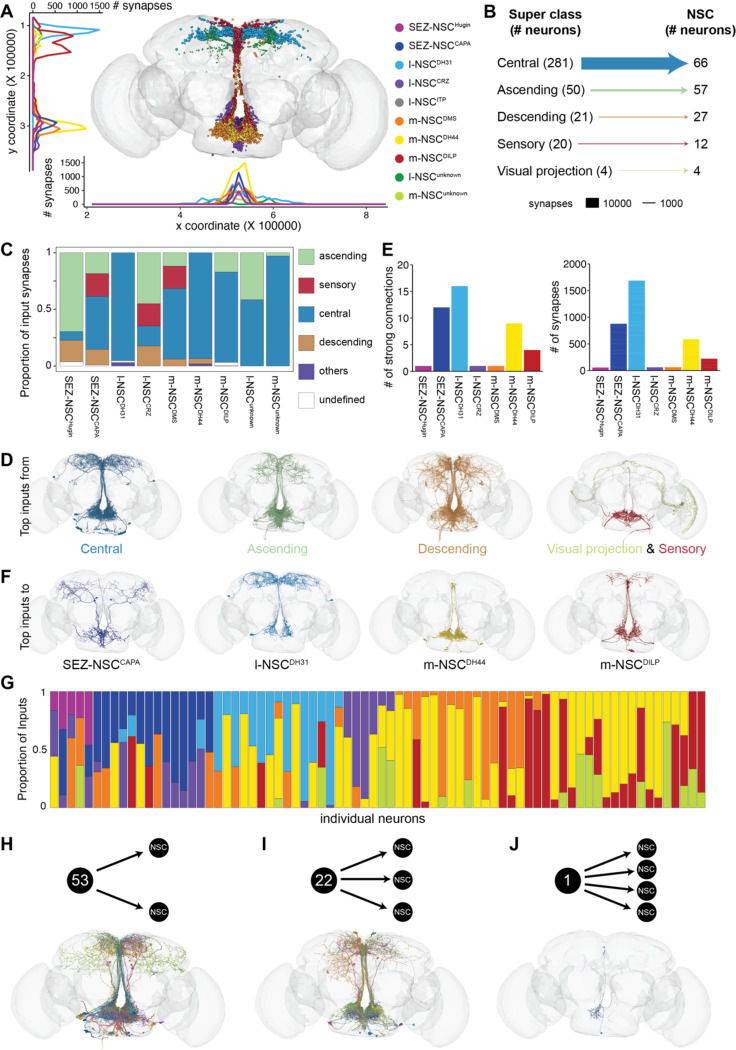
Synaptic inputs to NSC. **(A)** Postsynaptic sites of different NSC subtypes. Majority of the dendrites are found in the protocerebrum and SEZ. **(B)** Input to NSC grouped by the neuronal super classes annotated in the FlyWire connectome. Central neurons are the largest group providing inputs to NSC. **(C)** Proportion of inputs from various neuronal super classes to different types of NSC. **(D)** Reconstructions of neurons from different super classes which provide major inputs to NSC. Only the top 10 cell types per super class are shown. **(E)** Number of strong input connections (greater than 50 synapses) to each NSC subtype and the total number of synapses constituting these connections. **(F)** Reconstructions of neurons that provide major inputs (more than 50 synapses per connection) to SEZ-NSC^CAPA^, l-NSC^DH31^, m-NSC^DH44^ and m-NSC^DILP^. **(G)** Proportion of inputs from individual neurons to different NSC subtypes. In total, 76 neurons provide inputs to more than one type of NSC, with m-NSC^DH44^ receiving inputs from most of these neurons. Out of these 76 neurons, **(H)** 53 neurons provide inputs to two types of NSC, **(I)** 22 neurons provide inputs to three types of NSC and **(J)** 1 neuron provides input to four types of NSC. Reconstructions of corresponding neurons below each schematic. For (E) and (G), bars have been color coded according to the legend in the panel (A).

NSC represent a conduit through which information processed by the nervous system is relayed to peripheral tissues via different hormones. As such, several neural pathways are expected to converge onto NSC. To comprehensively elucidate the inputs to NSC, we first mapped the location of input synapses for each NSC type (Figure 3A and Figure 3 Supplement 1). Dendritic regions for the majority of NSC are found in the protocerebrum and SEZ. Interestingly, none of the 8 l-NSC^ITP^ had more than five synapses, which was the threshold used for identifying significant connections. Accordingly, their input synapses may be located outside the brain and/or the major inputs to these neurons are likely to be paracrine or hormonal in nature. We next examined the major neuronal classes providing inputs to NSC. Surprisingly, only 20 sensory neurons, all of which project to the SEZ, provide inputs despite most NSC having dendrites in that region (Figure 3B). This sensory input is directed to SEZ-NSC^CAPA^, l-NSC^CRZ^, and m-NSC^DMS^ (Figure 3C and Figure 3 Supplement 2A). Instead, a majority of the inputs to NSC arise from neurons in the central brain and ascending neurons from the ventral nerve cord (Figure 3D and Figure 3 Supplement 2A). l-NSC^DH31^, m-NSC^DH44^, and m-NSC^unknown^ almost exclusively receive inputs from central neurons (Figure 3 Supplement 2A). Interestingly, several classes of NSC receive inputs from descending neurons which are generally associated with the sensory-motor pathways that control locomotion and other behaviors. This suggests that descending neurons regulate both hormonal and motor output to synchronize physiological changes with appropriate behaviors, which could explain the inhibition of m-NSC^DILP^ during locomotion (Liessem et al., 2023). Lastly, l-NSC^DH31^ (not shown) receive small but significant direct input from ITP-expressing visual projection neurons that are part of the circadian clock network (Kurogi et al., 2023, Reinhard et al., 2024). Overall, m-NSC^DH44^ and m-NSC^DILP^ receive the largest number of inputs, and l-NSC^ITP^ and SEZ-NSC^Hugin^ receive the least synaptic inputs (Figure 3 Supplement 2B).

Next, we examined the neurotransmitters expressed in the neurons presynaptic to NSC (Figure 3 Supplement 3). For this, we used electron microscopy-based neurotransmitter predictions determined previously (Eckstein et al., 2024). We only focused on fast-acting neurotransmitters (i.e. acetylcholine, glutamate, and GABA) since those predictions were generally more reliable compared to other neurotransmitters such as serotonin (Eckstein et al., 2024). Both l-NSC^DH31^ and m-NSC^DH44^ receive strong glutamatergic inputs (Figure 3 Supplement 3A). Out of the three fast-acting neurotransmitters, GABA provides the least inputs (Figure 3 Supplement 3B) consistent with the proportional usage of the three neurotransmitters across the brain (Eckstein et al., 2024).

Since NSC receive inputs from several different cell types whose functions are yet unknown, we focused on cells that provide strong inputs to NSC. First, we examined the number of strong input connections (≥ 50 synapses) to each NSC subtype (Figure 3E). l-NSC^DH31^ receive the largest number of inputs via 16 strong connections and these are comprised of over 1500 synapses in total (Figure 3E). These inputs originate from neurons in the SEZ and the lateral horn (Figure 3F). SEZ-NSC^CAPA^, m-NSC^DH44^, and m-NSC^DILP^ also receive substantial inputs via strong connections (Figure 3E-F). In the case of SEZ-NSC^CAPA^, the strong presynaptic connections include sensory neurons and GABAergic olfactory projection neurons (Figure 3 Supplement 2A and Figure 3 Supplement 3A). For both m-NSC^DH44^ and m-NSC^DILP^, the strong inputs are exclusively from the SEZ. In summary, these results suggest that olfactory and gustatory pathways have a major effect on NSC activity and will be explored in detail later.

### Orchestration of physiology by multiple hormones

Several studies have previously characterized neuroendocrine pathways which regulate different aspects of *Drosophila* physiology including metabolism, reproduction, and osmotic homeostasis (Lee et al., 2015, Kubrak et al., 2016, Oh et al., 2019, Hadjieconomou et al., 2020, Koyama et al., 2021, Zandawala et al., 2021, Kurogi et al., 2023, Lee et al., 2023, Gera et al., 2024). Based on these and other studies, it is becoming increasingly evident that multiple hormonal systems interact to orchestrate specific physiological processes rather than individual hormones operating in isolation. For instance, DH31, DH44, CAPA, tachykinin, and ITP, which can all be released from brain NSC, influence osmotic homeostasis via direct actions on kidney-like Malpighian tubules (Halberg et al., 2015, Zandawala et al., 2018a, Agard et al., 2024, Gera et al., 2024). While these hormones could be released individually under specific contexts, we anticipate some of them to be co-released to elicit an additive or synergistic response (Zandawala et al., 2018a). As such, NSC producing these hormones could be regulated by common pre-synaptic partners. In total, we identified 76 neurons which provide inputs to more than one type of NSC, with m-NSC^DH44^ receiving inputs from most of these neurons (Figure 3G). This is in line with the role of DH44 in multiple processes including feeding, reproduction, metabolism, and osmotic homeostasis (Dus et al., 2015, Lee et al., 2015, Lee et al., 2023). Out of the 76 neurons, 53 neurons provide inputs to two types of NSC (Figure 3H) and 22 neurons provide inputs to three types of NSC (Figure 3I). In addition, one neuron (CB3500 cell type) influences four types of NSC (Figure 3J). Three other neurons of the CB3500 cell type are pre-synaptic to 2–3 NSC types. Therefore, CB3500 and other neurons that provide inputs to multiple NSC could potentially integrate information from various pathways to orchestrate the release of hormones in different combinations. Taken together, this analysis provides the basis to investigate the neural control of hormonal networks regulating various physiological processes.

### Characterizing sensory input pathways to NSC

**Figure 4:**
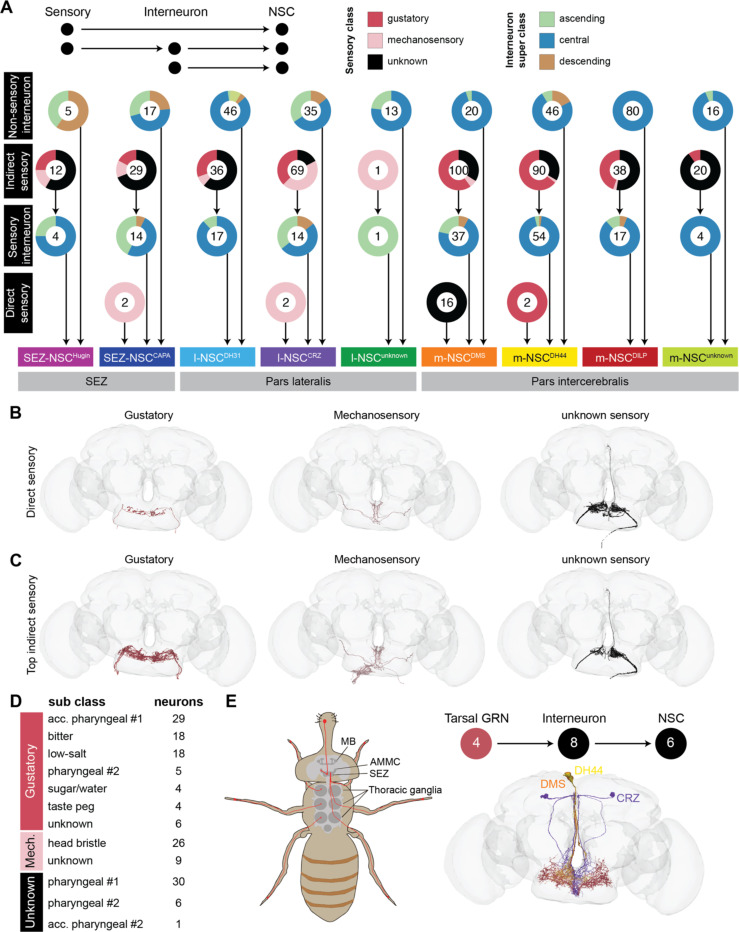
Sensory inputs to NSC. **(A)** Direct and indirect (disynaptic) sensory inputs to NSC. Interneurons mediating connectivity between sensory neurons and NSC are referred to as sensory interneurons. The donuts represent proportion of cells and the number in the donut reflects the total number of neurons in that group. NSC receive very minimal direct sensory inputs. Only gustatory, mechanosensory and unknown sensory inputs provide monosynaptic and disynaptic inputs to NSC. Note that l-NSC^ITP^ do not receive any significant synaptic inputs and are thus not represented here. **(B)** Reconstructions of sensory neurons (separated by class) providing direct inputs to NSC. **(C)** Reconstructions of sensory neurons providing indirect inputs to NSC. **(D)** Number of sensory neurons (grouped by sub class) that provide indirect inputs to NSC. **(E)** Schematic showing the projections of labellar and tarsal gustatory receptor neurons (GRN) from the periphery to the SEZ (adapted from (Freeman and Dahanukar, 2015)). Reconstructions of four tarsal GRN (colored red; classified as ascending neurons on Codex) that provide indirect inputs to six NSC (also shown). Abbreviations: acc. pharyngeal, accessory pharyngeal.

Sensory to endocrine pathways enable animals to maintain homeostasis by adjusting physiological processes in response to changing external environments. Since only 20 sensory neurons lie directly upstream of NSC (Figure 3B), we delineated both monosynaptic and disynaptic sensory-endocrine pathways in further detail. For simplicity, we refer to interneurons mediating these connections as sensory interneurons. By extension, neurons that provide inputs to NSC but don’t receive direct sensory input are called non-sensory interneurons (Figure 4A). Focusing first on the direct sensory input to NSC, only 2 gustatory receptor neurons (GRN) and 2 mechanosensory neurons are presynaptic to m-NSC^DH44^, SEZ-NSC^CAPA^, and l-NSC^CRZ^ respectively (Figure 4A-B). The remaining 16 sensory neurons, which have not yet been annotated, provide input to m-NSC^DMS^. These unknown sensory neurons project via the pharyngeal nerve to the SEZ and are likely involved in some aspect of feeding (Figure 4A-B).

Next, we examined indirect sensory inputs to each NSC type. Almost all types of NSC receive disynaptic inputs from GRN and mechanosensory neurons, with m-NSC^DMS^ and m-NSC^DH44^ receiving inputs from the largest number of neurons (Figure 4A and 4C). Although l-NSC^unknown^ is the second largest cluster after m-NSC^DILP^, they hardly receive any input from sensory neurons (Figure 4A). Looking further at the categories of sensory neurons, NSC receive inputs from all the major sub-classes of GRN as well as mechanosensory neurons in the head bristle (Figure 4D). The latter neurons likely regulate grooming behavior (Zhang et al., 2020); however, the link between grooming and endocrine signaling remains to be explored. Surprisingly, only 4 labellar sugar/water taste neurons are disynaptically connected to NSC. We expected sweet taste to be a major regulator of insulin, DH44, and CRZ signaling since these hormones have known roles in feeding and glucose homeostasis (Dus et al., 2015, Kubrak et al., 2016, Nässel and Zandawala, 2019). This prompted us to explore pathways from external GRN in other structures such as the legs (Figure 4E) (Freeman and Dahanukar, 2015). We identified 4 tarsal GRN in the connectome based on anatomical similarity (Thoma et al., 2016). Although tarsal GRN are not directly connected to NSC, they do provide indirect inputs to l-NSC^CRZ^, m-NSC^DH44^, and m-NSC^DMS^ (Figure 4E). Hence, tarsal and labellar GRN could regulate NSC activity to some extent. Intriguingly, the majority of the gustatory input to NSC stems from pharyngeal GRN (Figure 4D). This suggests that internal taste organs in the pharynx activated upon feeding rather than taste inputs alone from external GRN in the labellum and legs are more important for neurosecretion. Interestingly, gustatory, mechanosensory, and pharyngeal neurons are the only sensory neurons disynaptically upstream of NSC. Therefore, other sensory modalities such as vision and olfaction require additional layers of connectivity.

**Figure 5:**
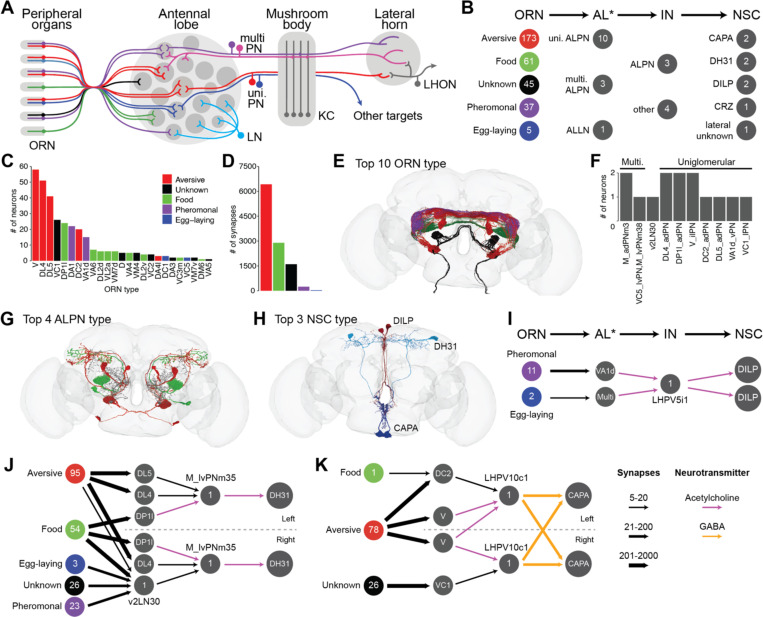
Olfactory inputs to NSC. **(A)** Schematic showcasing the flow of olfactory information from olfactory receptor neurons (ORN) in the antenna to the higher-order brain centers (e.g. mushroom bodies and lateral horn) via the antennal lobe (adapted from (Zhao and McBride, 2020)). **(B)** Number of neurons (grouped by different categories) that comprise the shortest pathway from ORN to NSC. ORN have been grouped based on their behavioral significance (based on (Zheng et al., 2022)). Antennal lobe associated neurons (AL*) include projection neurons (ALPN) and local interneurons (ALLN). IN represent interneurons that link AL* neurons and NSC. **(C)** Numbers of each ORN type that provide indirect inputs to NSC. **(D)** Number of synapses formed by these ORN. Note that the ORN which detect aversive odors followed by those that detect food odors provide the strongest indirect inputs to NSC. **(E)** Reconstructions of top ten ORN types. **(F)** Number of AL* in the pathway. v2LN30 is the only ALLN whereas the rest are ALPN. **(G)** Reconstructions of top four ALPN types and **(H)** top three NSC types that are part of this pathway. Bars in (C) and (D) and neurons in (E) and (G) have been colored based on their behavioral significance. **(I)** Pheromonal and egg-laying associated olfactory information is relayed to m-NSC^DILP^. **(J)** ORN belonging to all five behavioral categories provide inputs to l-NSC^DH31^. **(K)** SEZ-NSC^CAPA^ primarily receive aversive olfactory inputs. For I-K, the numbers within the circles indicate the number of neurons or the name of that neuron. Arrows have been weighted based on the number of synapses and colored based on the neurotransmitter mediating those connections (see legend). Abbreviations: LN, local interneuron; uni. PN, uniglomerular projection neuron; multi. PN, multiglomerular projection neuron; KC, Kenyon cell; LHON, lateral horn output neuron.

We next focused on olfactory pathways to NSC because pheromones and odors can have a profound impact on hormonal activity and resultant physiology (Lushchak et al., 2015, He et al., 2022, Zhang et al., 2022). For instance, acute exposure to food odors alone can trigger an anticipatory endocrine response (Lushchak et al., 2015). To obtain novel insights into olfactory modulation of NSC activity, we explored the shortest path from olfactory receptor neurons (ORN) to NSC. The canonical olfactory pathway in *Drosophila* and other insects begins with the ORN in the antennae and maxillary palps (Figure 5A). ORN of a given type all project to a single glomerulus in the antennal lobe. From here, uni-glomerular and multi-glomerular projection neurons (PN) transmit olfactory information to higher-order brain centers such as the mushroom body for learning and memory and the lateral horn which controls innate behaviors. Since NSC are not located within these brain regions, additional interneurons likely transmit the information from PN to NSC. In addition, local interneurons (LN) in the antennal lobes innervate multiple glomeruli and modulate olfactory pathways. Our analysis identified a total of 321 ORN which provide inputs to 8 NSC via this canonical pathway (Figure 5B). Moreover, we grouped the different ORN based on their behavioral significance (Zheng et al., 2022). ORN such as V, DL4 and DL5 that detect aversive odors comprise the largest group which provides input to NSC (Figure 5B-E). Food odor-related ORN such as DP1l, VA6, DL2d, VL2a, and VM7d make up the second largest group (Figure 5B-E). Olfactory information from the antennal lobe is relayed via 10 uni-glomerular PN and 3 multi-glomerular PN (Figure 5F and 5G). Interestingly, while all SEZ-NSC^CAPA^ are indirectly downstream of ORN, only 2 out of the 18 m-NSC^DILP^ and 2 out of the 6 l-NSC^DH31^ receive olfactory inputs (Figure 5H), further emphasizing the heterogeneity within these clusters. Intriguingly, only 11 pheromonal and 2 egg-laying related ORN provide input to m-NSC^DILP^ via this 3-hop pathway (Figure 5I). Pheromones likely trigger insulin release because both VA1d and LHPV5i1 that connect ORN to m-NSC^DILP^ are cholinergic. This pathway is distinct from a male-specific pathway identified earlier whereby pheromonal inputs from the leg ppk23 neurons activate m-NSC^DILP^ to inhibit courtship drive (Zhang et al., 2022). In addition, the strongest olfactory inputs are directed to l-NSC^DH31^ (Figure 5J). These inputs stem from all types of ORN, with aversive and food-related ORN providing the majority of the inputs. Since DP1l and M_lvPNm35 express the excitatory neurotransmitter acetycholine, food odors likely promote the release of DH31 and ITP from l-NSC^DH31^ (Gera et al., 2024). Since both hormones, albeit from different sources, have previously been implicated in feeding (Lin et al., 2022, Gera et al., 2024), l-NSC^DH31^ could also have a complementary role in feeding-related behaviors and physiological processes. Lastly, SEZ-NSC^CAPA^ also receive strong inputs from aversive ORN via cholinergic PN and GABAergic LHPV10c1 interneurons (Figure 5K). Although our analysis was based on the shortest 3-hop pathway, if we consider an additional layer of neurons and account for 4 hops, more than 91% of all ORN provide input to m-NSC^DILP^, l-NSC^DH31^ and SEZ-NSC^CAPA^ (not shown). At this level of connectivity, where most ORN are connected to NSC, it is difficult to decipher specific pathways. This is not surprising since the average shortest path length between any two neurons in the entire connectome is about 4 hops (Lin et al., 2024). In summary, olfactory inputs to NSC appear to be relatively sparse, and pheromonal and aversive odors seems to play a major role in hormonal signaling.

### Synaptic output pathways from NSC

**Figure 6:**
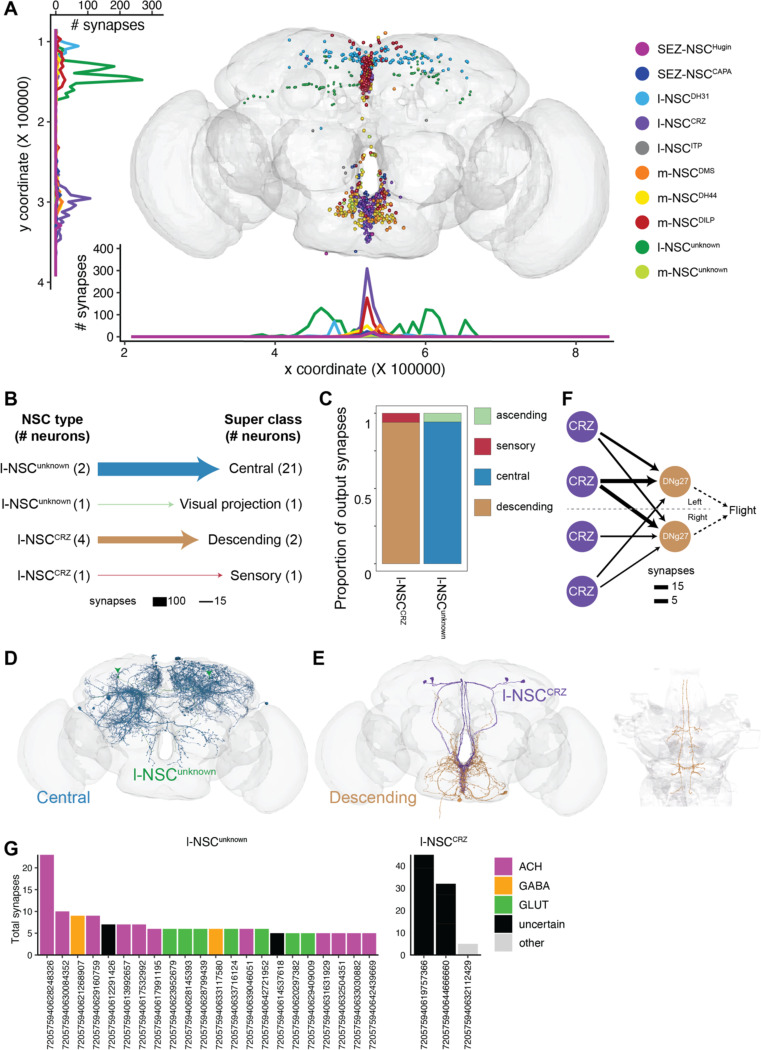
Synaptic output from NSC. **(A)** Presynaptic sites of different NSC subtypes. **(B)** Output from NSC grouped by the neuronal super classes annotated in the FlyWire connectome. Central neurons receive inputs from l-NSC^unknown^ and descending neurons receive inputs from l-NSC^CRZ^. **(C)** Proportion of outputs from different types of NSC to various neuronal super classes. **(D)** Reconstructions of l-NSC^unknown^ and all their postsynaptic partners. **(E)** Reconstructions of l-NSC^CRZ^ and all their postsynaptic partners (descending neurons). The descending neurons primarily innervate the wing tectulum. **(F)** Weighted connections between l-NSC^CRZ^ and DNg27 descending neurons which innervate the wing tectulum and could thus regulate flight. **(G)** Individual postsynaptic partners of l-NSC^unknown^ and l-NSC^CRZ^ sorted based on the number of synapses and colored based on their neurotransmitter identity.

While NSC predominantly release hormones into the circulation following their activation, some NSC types can also signal to other neurons within the brain (King et al., 2017). With this in mind, we examined synaptic output from NSC. Most NSC do in fact form output synapses within the brain, with l-NSC^CRZ^ and l-NSC^unknown^ having the largest number (Figure 6A and Figure 6 Supplement 1). Similar to the location of their dendrites, these output synapses are situated in the protocerebrum and SEZ. Although all types of NSC form output synapses, most of these comprise a connection which does not meet our threshold of 5 synapses. Hence, l-NSC^CRZ^ and l-NSC^unknown^ are the only NSC types which provide significant output to other neurons (Figure 6B). The output from l-NSC^unknown^ is primarily directed to cholinergic and glutamatergic central neurons (Figure 6B-D and Figure 6G) whereas two pairs of l-NSC^CRZ^ provide strong output to DNg27 descending neurons (Figure 6B-C and Figure 6E-F). DNg27, whose function is yet unknown, innervate the wing neuropil so they could potentially modulate flight (Figure 6E-F). We also explored the output from NSC after lowering the threshold of significant connections to 2 synapses (Figure 6 Supplement 2). Although the output from NSC increases substantially at this threshold, most of this output is directed to undefined cells which include partial fragments as well as non-neuronal cells (Figure 6 Supplement 2A-C). Nonetheless, additional output to central, endocrine, and descending neurons is also observed, some of which could be biologically significant even though only a few synapses mediate these connections. In conclusion, sparse synaptic output from NSC agrees with the expectation that they mainly signal in a paracrine and endocrine manner.

### Identifying the molecular basis of paracrine and hormonal NSC output pathways

**Figure 7:**
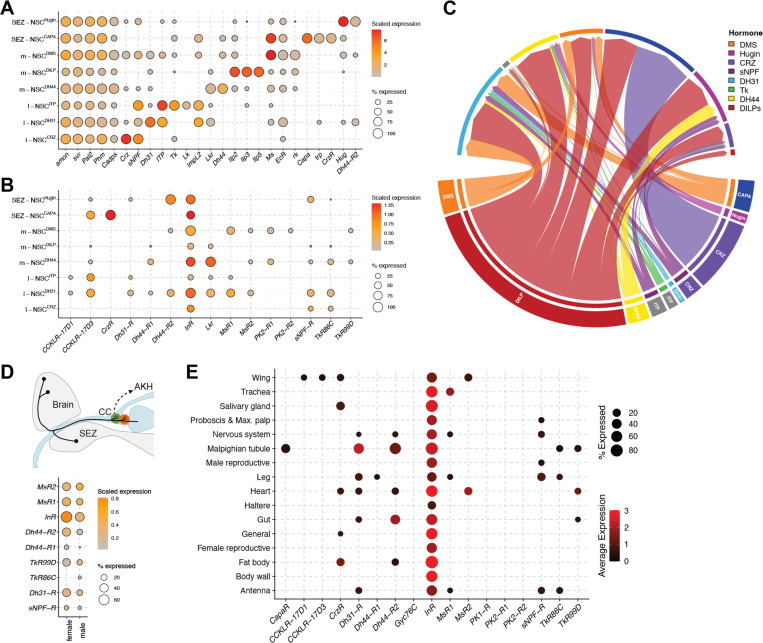
NSC interconnectivity and endocrine output. **(A)** Identification of single-cell transcriptomes representing different NSC subsets in the adult brain (Davie et al., 2018). All NSC express genes required for neuropeptide processing and release (*amon*, *svr*, *Pal2*, *Phm* and *Cadps*) and were identified primarily based on the neuropeptides that they express. **(B)** Dot plot showing expression of receptors in NSC. Expression of only those receptors whose corresponding neuropeptides are expressed in NSC are shown. **(C)** Connectivity diagram (weighted based on neuropeptide and receptor expression) showing putative paracrine connectivity between different types of NSC. Note that short neuropeptide F (sNPF) and myosuppressin (DMS) are expressed in two different NSC subtypes. Ion transport peptide and CAPA pathways are not included because their receptors were not detected in these transcriptomes. Leucokinin was excluded because its expression levels were below the threshold used here. Dot plot showing the expression of neuropeptide receptors in **(D)** adipokinetic hormone cells of the corpus cardiacum and **(E)** all the tissues in adults. “General” in panel **E** includes cell types that are found across multiple tissues including sensory neuron, visceral muscle and hemocytes amongst others. See Figure 7 Supplement 3 for all the different cell types that are part of this cluster.

The availability of large-scale single-cell transcriptome datasets can now enable us to identify and explore transcriptomes of rare cell types such as NSC. We have recently used this strategy to determine the modulatory inputs to m-NSC^DILP^ (Held et al., 2024), l-NSC^ITP^ (Gera et al., 2024), and some other NSC types (Reinhard et al., 2024). Here, we expand this approach to first identify single-cell transcriptomes of all NSC types based on previously established markers (Figure 7A). Consistent with our anatomical mapping (Figure 2D), *Capa* and *Ms* are co-expressed in SEZ-NSC^CAPA^. Given the proximity of all NSC axon terminations, it is extremely likely that a hormone released from a given NSC will influence the activity of other NSC types if its receptor is expressed in those cells. In fact, we have previously shown CRZ to inhibit CAPA release from a different set of neurosecretory cells much further away in the ventral nerve cord (Zandawala et al., 2021). Therefore, we examined the expression of hormone receptors in all the NSC transcriptomes to determine the molecular substrates for paracrine interaction between different NSC types (Figure 7B). Consistent with previous studies, *CrzR* is expressed in SEZ-NSC^CAPA^ (Zandawala et al., 2021), *sNPF-R* and *Lkr* in m-NSC^DILP^ (Kapan et al., 2012, Zandawala et al., 2018c) and *Dh44-R2* in SEZ-NSC^Hugin^ (Mizuno et al., 2021). While receptors for CAPA and ITP were not detected in any transcriptomes, receptors for other hormones were expressed in varying amounts. l-NSC^DH31^ express receptors for several hormones and thus appear to be heavily modulated (Figure 7B). Further, *InR* (insulin receptor) is expressed in all cell types consistent with the role of insulin in promoting cellular glucose uptake (O’Neill, 2013). Having mapped the expression of hormones and their receptors in different types of NSC, we sought to determine the strength of putative paracrine connections based on their expression levels. Thus, higher expression of both the hormone and receptor implies a stronger connection. Using this approach, we show the extent of putative paracrine connectivity between different hormonal systems (Figure 7C and Figure 7 Supplement 1). We used a conservative approach to reduce false prediction by setting a stringent expression threshold for the hormone levels. Hence, only those hormones which were expressed in at least 50% of the cells in each cluster were considered to be present (Figure 7 Supplement 1I). Our analysis reveals that paracrine signaling can greatly enhance the interactions between different hormonal pathways.

Since all NSC have their release sites on or near adipokinetic hormone (AKH) producing cells of the CC, various hormones can influence the release of AKH (Oh et al., 2019, Koyama et al., 2021). Glucagon-like AKH, along with DILPs, is a major regulator of metabolic homeostasis and associated behaviors (Nässel and Zandawala, 2019). Consequently, modulation of AKH release is one way of regulating metabolic physiology. We thus examined single-cell transcriptomes of AKH cells for expression of hormone receptors (Figure 7D). While sNPF-R expression in AKH cells was demonstrated previously (Oh et al., 2019), we additionally show the presence of receptors for DMS, DH44, DH31 and tachykinin. Thus, these neuropeptides could regulate metabolic homeostasis via AKH-signaling in addition to their known roles in feeding-related processes (Dus et al., 2015, Nässel and Zandawala, 2019, Hadjieconomou et al., 2020).

Previously, Nässel and Zandawala (2019) had catalogued the expression of neuropeptide receptors across all the *Drosophila* tissues. However, that analysis was based on a microarray-based dataset (Chintapalli et al., 2007) and therefore lacks the sensitivity and resolution offered by current sequencing technologies. We sought to fill this gap by cataloguing the expression of hormone receptors using Fly Cell Atlas, a single-cell transcriptomic resource of all cells of the adult fly. Expression of hormone receptors in salivary glands, nervous system, Malpighian tubules, heart, gut and fat body (Figure 7E and Figure 7 Supplement 2-4) is largely consistent with the previous analysis. For instance, expression of *CapaR*, *Dh31-R* and *Dh44-R2* in Malpighian tubules and *CrzR*, *Dh31-R*, *Dh44-R2* and *TkR99D* in the heart was reported previously (Nässel and Zandawala, 2019). Interestingly, we now additionally detect *TkR86C* and *TkR99D* expression in the Malpighian tubules and *MsR2* expression in the heart. Examining expression of these receptors at cellular resolution reveals that *TkR99D* is highly expressed in stellate cells of the Malpighian tubules and *MsR2* is strongly expressed in the alary muscles of the heart (Figure 7 Supplement 3). Insights from a similar analysis were recently used to characterize the effects of tachykinin on stellate cells (Agard et al., 2024) and our analysis here suggests that DMS can modulate heart contractility via activation of *MsR2* on the alary muscles. Hence, this approach can uncover novel cellular targets of various hormones. Importantly, Fly Cell Atlas also includes tissues such as trachea, leg, wing, haltere, proboscis and maxillary palp where expression of hormone receptors has not been explored comprehensively. We reveal expression of several receptors including *CrzR*, *MsR1*, *MsR2*, *Dh44-R1* and *Dh44-R2* in sensory neurons of the antenna and other tissues (Figure 7 Supplement 3). Thus, m-NSC^DMS^, m-NSC^DH44^, l-NSC^CRZ^ not only receive direct sensory inputs (Figure 4A) but they could also modulate other types of sensory neurons, forming sensory-endocrine feedback loops. Taken together, our analysis presents an important resource to functionally characterize novel hormonal targets.

## Discussion

Here, we describe the first connectome of a neurosecretory network in an adult animal brain. This connectome is based on 80 NSC which can be subclassified into 10 categories based on neuropeptide expression, morphological similarity and/or synaptic connectivity. Moreover, our integration of connectomics with anatomical analyses provides a comprehensive view of the NSC landscape and their connectivity in the adult *Drosophila* brain. Our analyses reveal a functionally diverse, yet highly interconnected neuroendocrine system which provides the basis to perform comparisons with other neuroendocrine connectomes from larval *Platynereis* and *Drosophila* established previously.

### Comparison of neuroendocrine connectomes across development and species

The composition of the adult NSC is different compared to larvae. We observed an expansion of m-NSC^DILP^ and m-NSC^DMS^ clusters along with the presence of two additional populations of putative NSC whose identity and function remains to be explored. The expansion in the number of identified NSC subtypes from larvae to adults underscores developmental changes that might reflect the life-history adaptation of these cells to new physiological demands or environmental challenges. For instance, adults are considerably larger than larvae and comprised of more cells in general. Glucose uptake by these cells is modulated by DILPs via InR. Hence, additional m-NSC^DILP^ may be needed for increased DILP production and release to compensate for this size increase. The increase in NSC number is not merely a quantitative change but suggests functional diversification, as evident by the presence of previously unidentified m-NSC and l-NSC populations in adults. Since the connectome dataset examined here does not include the axonal projections of these neurons outside the brain volume, it is unclear which regions they innervate. However, given their smaller size compared to other NSC populations, they might influence the release of other hormones via local actions at neurohemal sites rather than hormonal regulation of peripheral tissues.

Similar to the *Drosophila* and *Platynereis* larval neuroendocrine connectomes, NSC in adult *Drosophila* have limited synaptic output in the brain (Williams et al., 2017, Huckesfeld et al., 2021). Eclosion hormone-expressing NSC are the only cells with synaptic output in larvae. However, these cells undergo apoptosis soon after eclosion and are thus not found in mature adults. In contrast, l-NSC^CRZ^ and l-NSC^unknown^ provide the majority of synaptic output. l-NSC^CRZ^ are especially of interest here because only 4 out of the 6 neurons in this cluster lie upstream of DNg27 descending neurons that primarily innervate the wing neuropil. The remaining two l-NSC^CRZ^ are internal glucose sensors which signal via sNPF to influence DILP and AKH release, which in turn regulate glucose homeostasis (Oh et al., 2019). Furthermore, DILPs suppress starvation-induced food search whereas AKH promotes this behavior (Yu et al., 2016). Therefore, it is plausible that one subset of l-NSC^CRZ^ affect starvation-dependent locomotor activity via AKH and DILPs, while the other subset modulate flight via DNg27 in response to starvation. This circuit motif is only found in adults and likely accounts for different locomotion strategies across development. In addition to l-NSC^CRZ^, other NSC types including m-NSC^DILP^, m-NSC^DH44^ and l-NSC^DH31^ also exhibit heterogeneity in their morphology, synaptic inputs, and gene expression. This heterogeneity may reflect an adaptive mechanism allowing for fine-tuned responses to environmental and physiological cues. In support of this, only a subset of m-NSC^DILP^ and m-NSC^DH44^ express the mechanosensitive channel Piezo (Wang et al., 2020, Oh et al., 2021). Similar functional differences between l-NSC^DH31^ subtypes remain to be explored.

Regarding non-synaptic output, neuropeptides expressed in the *Platynereis* neuroendocrine center are largely distinct from those found in the *Drosophila* brain NSC, with insulin-like peptides, tachykinin and sulfakinin being the only hormones that are common across both species (Williams et al., 2017, Huckesfeld et al., 2021). The latter was previously shown to be expressed in a subset of m-NSC^DILP^ (Soderberg et al., 2012); however, we were unable to detect it in our single-cell transcriptomic analysis, likely due to low expression. Interestingly, cholecystokinin, the vertebrate ortholog of sulfakinin, is also expressed in the hypothalamus (Williams et al., 2017, Nässel and Wu, 2022). Thus, the expression of cholecystokinin/sulfakinin in neuroendocrine centers and their function in regulating satiety are conserved across evolution. Examination of neurosecretory cells in other species can shed light on other conserved hormonal systems.

### Sensory inputs to NSC

Our analysis of sensory input pathways revealed that only 20 sensory neurons, primarily gustatory and mechanosensory neurons, provide direct inputs to NSC. While this number is much smaller than the corresponding number in larvae (Huckesfeld et al., 2021), that study utilized a synaptic threshold of only one. Perhaps one synapse may be sufficient to modulate the activity of NSC on slower timescales. However, this number also includes some transient connections which may not persist due to context-dependent synaptic plasticity. Therefore, we used a higher threshold in line with other studies using the same dataset (Dorkenwald et al., 2023, Reinhard et al., 2024). Our results indicate that SEZ-NSC^CAPA^ receive and integrate inputs from both mechanosensory and olfactory pathways. This cross-sensory integration allows an animal to comprehensively assess the environment such as during odor-guided navigation where both wind and odor inputs are important. These sensory modalities could also be important during feeding to assess food quality and texture. Our olfactory circuit tracing analysis also revealed strongest olfactory inputs to l-NSC^DH31^. Interestingly, l-NSC^DH31^ respond to both pheromonal and food-odor inputs. Integration of these odors can attract flies of both sexes to food sources that are suitable for mating (Kohl et al., 2015). Based on this circuit motif, we would expect these cells to play a role in feeding, mating and/or courtship. Consistent with this prediction, DH31 from these neurons targets the CA to suppress juvenile hormone signaling (Kurogi et al., 2023), which in turn influences egg maturation, courtship and sex pheromone production (Bilen et al., 2013). l-NSC^DH31^ also express ITP which is important for feeding and metabolic homeostasis (Gera et al., 2024). Moreover, l-NSC^DH31^ could thus be part of a feedback loop where they receive pheromonal and food-related odor inputs to regulate pheromone production and food intake. Overall, these findings highlight the vital role of l-NSC^DH31^ in integrating sensory information to regulate crucial interdependent behaviors.

### Interactions between hormonal pathways

Our analyses highlight extensive interactions between hormonal systems via both their synaptic inputs and hormonal output. Multiple NSC types receive inputs from the same set of pre-synaptic neurons, indicating that different hormonal pathways do not operate in isolation but rather interact within a complex network. m-NSC^DH44^ are of particular interest here since they receive the most extensive input from neurons which also provide inputs to other types of NSC. Consequently, circuits which influence other hormones could also affect DH44 release. In addition, m-NSC^DH44^ can function as cell-autonomous glucose and amino acid sensors (Dus et al., 2015, Yang et al., 2018). m-NSC^DH44^ in females can also integrate inputs regarding the quality of their male partner’s ejaculate by directly sensing a phospho-galactoside present in the male ejaculate (Kim et al., 2024). Further, these cells are also intrinsically mechanosensitive and can monitor the feeding state of the animal based on crop distension (Oh et al., 2021). Thus, various inputs regulate the activity of m-NSC^DH44^ and DH44 could act as a major co-coordinator of physiology and behavior. For instance, DH44 could be co-released with DMS and/or DILPs to orchestrate feeding and reproductive processes (Nässel and Zandawala, 2019, Hadjieconomou et al., 2020). In addition to the regulation of hormonal pathways by common synaptic inputs, the various hormones can also modulate each other’s release via paracrine signaling. Hence, sulfakinin and DILPs from m-NSC^DILP^ can interact with other signaling pathways as their receptors are expressed in most NSC types. Moreover, l-NSC^DH31^ and AKH-producing cells are also heavily modulated by different hormones. Therefore, in agreement with their major roles in metabolic physiology, several endocrine pathways converge on m-NSC^DILP^ (Held et al., 2024) and AKH-producing cells.

### Limitations of our approach

Our connectomics and transcriptomics-based approach to decipher synaptic and paracrine connectivity of the NSC has some general limitations. Firstly, the novel pathways identified here are only putative until experimentally verified using functional connectivity or behavioral analyses. Moreover, our analyses underestimate the connectivity for several reasons: 1) the algorithm used for synapse prediction was not 100% effective, 2) we used a fairly stringent threshold (≥ 5 synapses) for assessing connectivity, 3) some small neuronal fragments have not yet been proofread and 4) NSC could form synapses with each other near their release sites and outside the brain volume examined here. Moreover, NSC can also couple electrically via gap junctions which are not accounted for here (Orchard and Shivers, 1986, Alvarado Alvarez et al., 1993). Lastly, the connectome depicts a static snapshot of connectivity which we anticipate changing with age as well as with the mating and feeding status of the animal.

### Conclusion and future directions

Future research should focus on elucidating the functional implications of the observed connectivity patterns, particularly how specific sensory inputs to NSC are translated into physiological responses. Moreover, characterization of the neuroendocrine connectome in a male brain can provide insights into sexual dimorphism within the neuroendocrine pathways. Taken together, our comprehensive characterization of the adult *Drosophila* neuroendocrine network connectome provides a foundation to experimentally disrupt endocrine pathways and establish causal relationships with disorders such as diabetes, hypertension, and infertility. In addition, it provides a blueprint for understanding complex hormonal networks and how they orchestrate animal behaviors and physiology.

## Materials and methods

### Fly strains

*Drosophila melanogaster* strains used in this study are listed in Supplementary Table 1. Fly lines were obtained from the Bloomington *Drosophila* Stock Center (BDSC). Flies were reared at 25°C under LD12:12 on a standard *Drosophila* medium containing 8.0% malt extract, 8.0% corn flour, 2.2% sugar beet molasses, 1.8% yeast, 1.0% soy flour, 0.8% agar and 0.3% hydroxybenzoic acid.

### Immunohistochemistry and confocal imaging

Immunostainings were performed as described previously (Gera et al., 2024). Briefly, whole female flies were fixed in 4% paraformaldehyde in phosphate-buffered saline (PBS) with 0.5% Triton X-100 (PBS-T) for 2.5 h on a nutator at room temperature (RT). Fixed flies were washed four times with PBS-T before dissecting their brains. The samples were blocked in PBS-T containing 5% normal goat serum for 1 hour at RT and subsequently incubated in primary antibodies at 4°C for 48 h. Following four washes with PBS-T, the brains were incubated in secondary antibodies at 4°C for 48. Lastly, the samples were washed four times in PBS-T and mounted using either Fluoromount-G^™^ (Invitrogen, Thermo Fisher) or Vectashield mounting medium (Vector Laboratories, Burlingame, CA, USA). Images were acquired using Leica SPE and SP8 confocal microscopes (Leica Microsystems) using 20x or 40x objectives.

All the primary and secondary antibodies are listed in Supplementary Table 2.

### Connectome datasets and NSC identification

We used the v783 snapshot of the FlyWire whole brain connectome and its annotations (annotations last updated: 12 June 2024) for all the analyses (Dorkenwald et al., 2023, Schlegel et al., 2023). To identify NSC in the FlyWire connectome, we first identified NSC subsets that have a characteristic morphology and location, e.g. l-NSC^ITP^. We then identified the nerve bundle (nervii corpora cardiaca, NCC) through which l-NSC^ITP^ axons exit the brain. This nerve bundle contains all the axons for NSC in the *Drosophila* brain. We manually assessed other axons in NCC to identify the remaining NSC in the connectome. Independently, we also examined the cross-section of the soma of all putative NSC for the presence of dense core vesicles, which suggests that they contain neuropeptides/neuromodulators.

FlyWire cell IDs of identified NSC are provided in Supplementary Table 3.

### Neurotransmitter predictions

Neurotransmitter predictions are based on Eckstein et al. (2024). We only considered neurotransmitter prediction scores for fast-acting neurotransmitters (i.e., acetylcholine, glutamate, and GABA) that were greater than 62%.

### Data visualization

Data was visualized using ggplot2 (v 3.5.1, Wickham, 2016) and circlize (v 0.4.16) for R (v 4.4.1) in RStudio (2024.04.2+764) (Gu et al., 2014). NSC reconstructions were downloaded using the navis library (v 1.0.4, https://github.com/navis-org) and cloud-volume library (v 8.10.0, https://github.com/seung-lab/cloud-volume) for python (v 3.8.5), and visualized using blender (v 3.01, Community, B. O. 2018). All other neuron reconstructions were visualized using FlyWire neuroglancer (Dorkenwald et al., 2022).

### Connectivity analyses

Natverse libraries (v 0.2.4) for R in RStudio (Bates et al., 2020) were used to analyze the connectivity data. NSC were clustered based on all their synaptic inputs with coconatfly (v 0.1.0.9000) for R (Schlegel et al., 2023). Filtered synapses were retrieved in Python using the navis and pandas (v 1.1.3) libraries. Unless stated otherwise, a threshold of 5 synapses was used to determine significant connections. Our analyses were based on a custom code generated previously (Reinhard et al., 2024).

### Prediction of paracrine and endocrine networks

Single-cell transcriptomes of AKH-producing cells and all tissues (stringent version) were obtained from the Fly Cell Atlas (Li et al., 2022). We manually reclassified the cell clusters for different tissues since some tissues included artefacts or cells that are not unique to a particular tissue (e.g., hemocytes). Cell types present in multiple tissues were classified as “general”. Unannotated and artefact clusters were excluded. Head and body clusters were also excluded since they included cell types that were present in individual tissues.

NSC transcriptomes were identified from the brain single-cell transcriptomes generated previously (Davie et al., 2018). The parameters used to identify the different NSC types were based on previous studies and provided below (Kahsai et al., 2010, Kapan et al., 2012, Miyamoto and Amrein, 2014, Cannell et al., 2016, Yang et al., 2018, Nässel and Zandawala, 2019, Oh et al., 2019, Hadjieconomou et al., 2020, Mizuno et al., 2021, Zandawala et al., 2021, Gera et al., 2024).

l-NSC^DH31^ (6 cells): ITP > 2 & Dh31 > 4 & amon > 0 & Phm > 0

l-NSC^CRZ^ (4 cells): Crz > 3 & sNPF > 3 & Dh44 == 0 & ITP == 0 & ChAT == 0 & Gr64a == 0 & Phm > 0

l-NSC^ITP^ (7 cells): Tk > 1 & sNPF > 1 & ITP > 1 & ImpL2 > 1 & Crz == 0

m-NSC^DH44^ (6 cells): Dh44 > 2 & CG13248 > 0 & CG13743 > 0 & Lkr > 0 & Phm > 0

m-NSC^DILP^ (14 cells): Ilp2 > 3 & Ilp3 > 3 & Ilp5 > 3 & ChAT == 0

m-NSC^DMS^ (5 cells): Ms > 2 & EcR > 0 & rk > 0 & amon > 0 & Phm > 0

SEZ-NSC^CAPA^ (3 cells): Capa > 0 & CrzR > 0 & trp > 0 & amon > 0 & Phm > 0

SEZ-NSC^Hugin^ (15 cells): Hug > 3 & Dh44-R2 > 0 & amon > 0 & Phm > 0

To determine the strength of paracrine connections between NSC subtypes, we multiplied expression scores (see below for calculation) of a neuropeptide with that of its corresponding receptor. Some neuropeptides mediate their effects via two receptors. If both receptors were expressed in a given cell-type, we only considered the one with higher expression for the sake of simplicity. To reduce false positives, only those neuropeptides which were expressed in at least 50% of the cells in each cluster were considered to be present. In the case of receptors, we used a percent expression threshold of 5%. Since m-NSC^DILP^ produce DILP2, DILP3, and DILP5, all of which target the same receptor, we used their average expression for all analyses. We calculated an expression score by multiplying the scaled expression of a gene with the percent of cells expressing that gene. Additionally, we filtered neuropeptide expression scores below 2.5%.

All analyses were performed in R-Studio (2024.04.2+764) using the Seurat package (v4.4.0 (Hao et al., 2021)).

## Supplementary Material

Supplement 1

Supplement 2

## Figures and Tables

**Table 1:** Classification of *Drosophila* neurosecretory cells (NSC) based on their cell body position in the central brain

NSC Super Class	Neuropeptide	Other common names	NSC Class	# in larvae	Expected # in adults	Observed # in adults
Medial	Myosuppressin (DMS)	SP3	m-NSC^DMS^	4	4	6
Medial	Diuretic Hormone 44 (DH44)	DH44-PI	m-NSC^DH44^	6	6	6
Medial	*Drosophila* Insulin-Like Peptides (DILP) 2, 3 and 5	IPC	m-NSC^DILP^	14	14	18*
Medial	Eclosion Hormone (EH)	V_m_	NSC^EH^	2	0	0
Lateral	Corazonin (CRZ)	DLP, CC-LP2	l-NSC^CRZ^	6	14	6
Lateral	Ion-Transport Peptide (ITP)	ALK, ipc-1	l-NSC^ITP^	8	8	8
Lateral	Diuretic Hormone 31 (DH31)	CA-LP	l-NSC^DH31^	6	6	6
Lateral	ProThoracicoTropic Hormone (PTTH)	PG-LP	l-NSC^PTTH^	4	0	0
Subesophageal	Capability (CAPA)	Large SEG neurons	SEZ-NSC^CAPA^	2	2	2
Subesophageal	Hugin	HugRG	SEZ-NSC^Hugin^	4	4	4
Medial	Unknown		m-NSC^unknown^			10
Lateral	Unknown		l-NSC^unknown^			14
		**Total**		56	58	80

* **Notes:** It is unclear at this point if all 18 m-NSC^DILP^ express all three or different combinations of DILP2, 3 and 5.

## Data Availability

Connectivity analysis can be performed using the cell IDs provided at https://codex.flywire.ai/.
