# Supplementary material for: Synaptic connectome of a neurosecretory network in the Drosophila brain
Source: bioRxiv. 2024 Aug 29:2024.08.28.609616. Preprint. [Version 1] doi: 10.1101/2024.08.28.609616 (PMC11384003; doi:10.1101/2024.08.28.609616)
Supplement: Supplement 2 [file NIHPP2024.08.28.609616v1-supplement-2.pdf]

bioRxiv preprint doi: https://doi.org/10.1101/2024.08.28.609616; this version posted August 29, 2024. The copyright holder for this preprint (which was not certified by peer review) is the author/funder, who has granted bioRxiv a license to display the preprint in perpetuity. It is made available under aCC-BY 4.0 International license.

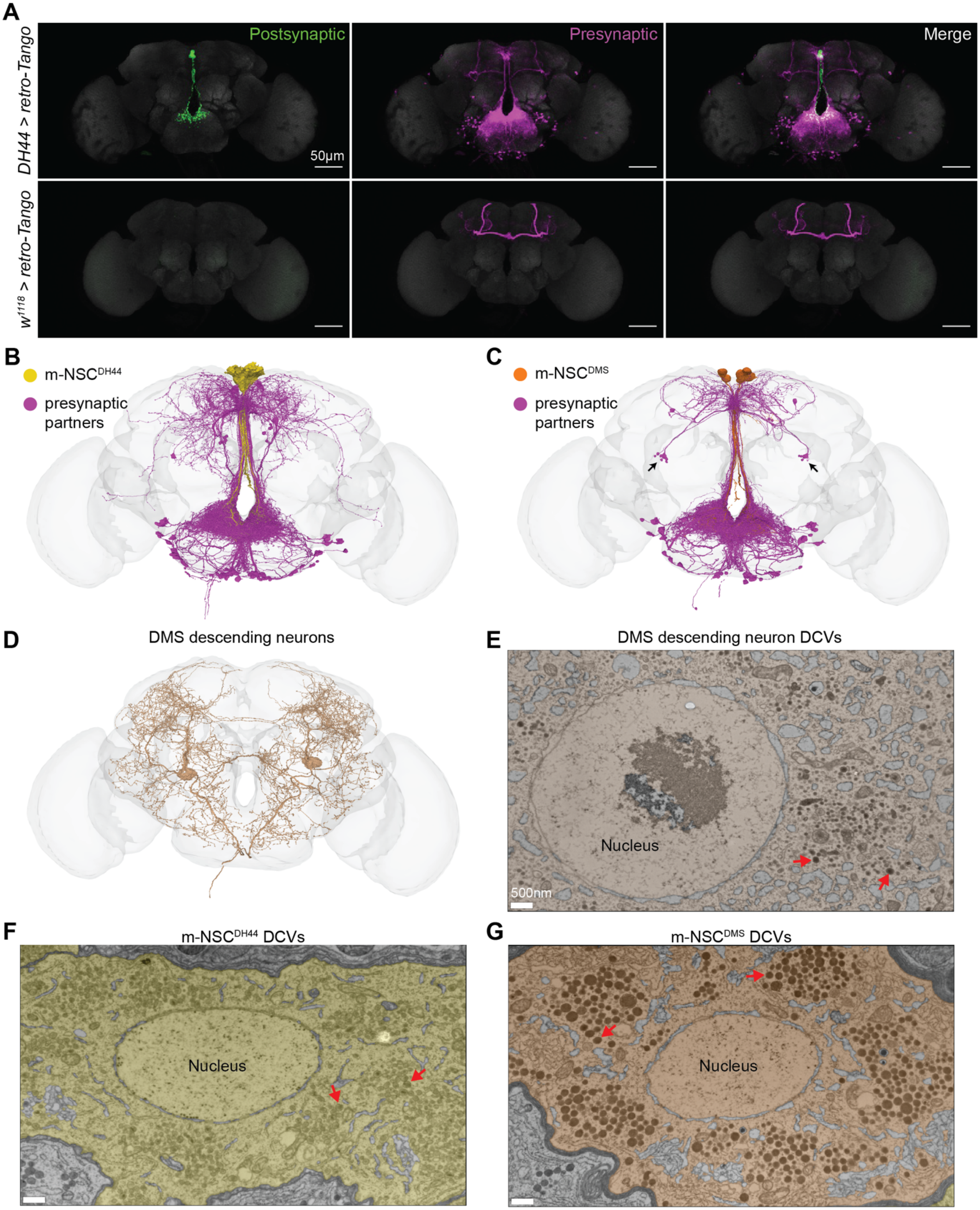

bioRxiv preprint doi: https://doi.org/10.1101/2024.08.28.609616; this version posted August 29, 2024. The copyright holder for this preprint (which was not certified by peer review) is the author/funder, who has granted bioRxiv a license to display the preprint in perpetuity. It is made available under aCC-BY 4.0 International license.

Figure 1 Supplement 1: Differences between m-NSC^{DH44} and m-NSC^{DMS}. (A) Retrograde trans-synaptic labelling of m-NSC^{DH44}. m-NSC^{DH44} are labelled in green and their presynaptic partners are labelled in magenta. Note the ectopic expression in mushroom body which is also visible in the controls. *In silico* retrograde tracing of **(B)** m-NSC^{DH44} and **(C)** m-NSC^{DMS}. Both of these NSC subtypes receive majority of their inputs from neurons in the SEZ which have similar location and morphology. However, m-NSC^{DMS} also receive inputs from a group of central neurons (marked with an arrow) that are not visible in (A) and (B). **(D)** Reconstruction of myosuppressin (DMS) descending neurons. Electron micrographs showing a cross section of **(E)** DMS descending neuron, **(F)** m-NSC^{DH44} and **(G)** m-NSC^{DMS} cell soma. Both types of DMS-expressing cells have darker dense core vesicles (marked by red arrows) compared to those found in m-NSC^{DH44}.

bioRxiv preprint doi: https://doi.org/10.1101/2024.08.28.609616; this version posted August 29, 2024. The copyright holder for this preprint (which was not certified by peer review) is the author/funder, who has granted bioRxiv a license to display the preprint in perpetuity. It is made available under aCC-BY 4.0 International license.

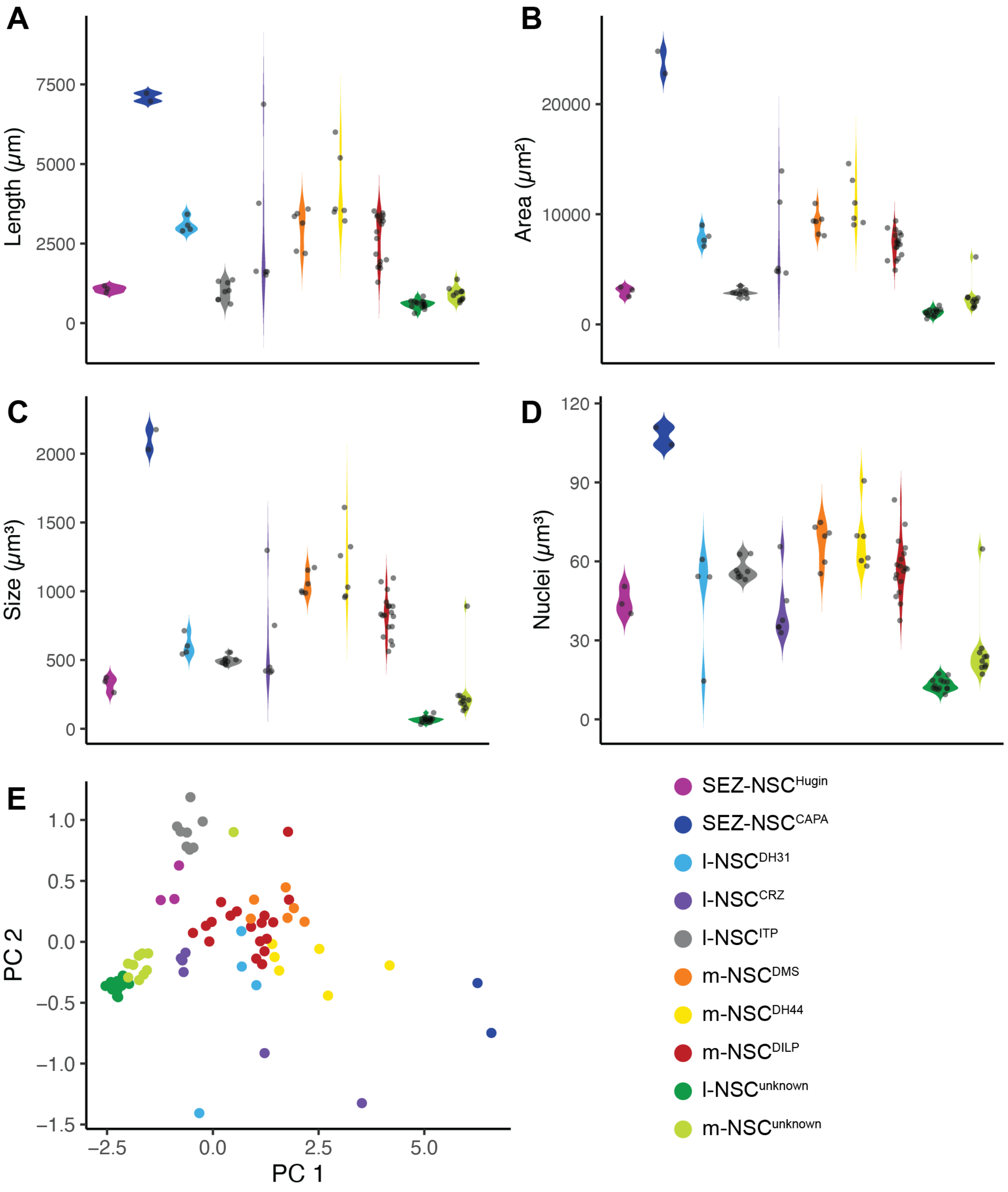

Figure 1 Supplement 2: Morphological characteristics of NSC. (A) cable length, **(B)** surface area, **(C)** cell volume and **(D)** nuclei volume of different NSC subtypes. **(E)** Principal component analysis of these four features reveals that the NSC of a given subtype generally cluster together. Note the high variability for l-NSC^{CRZ}, l-NSC^{DH31}, m-NSC^{DH44} and m-NSC^{DILP} populations, suggesting that they comprise of morphologically heterogenous subpopulations.

bioRxiv preprint doi: https://doi.org/10.1101/2024.08.28.609616; this version posted August 29, 2024. The copyright holder for this preprint (which was not certified by peer review) is the author/funder, who has granted bioRxiv a license to display the preprint in perpetuity. It is made available under aCC-BY 4.0 International license.

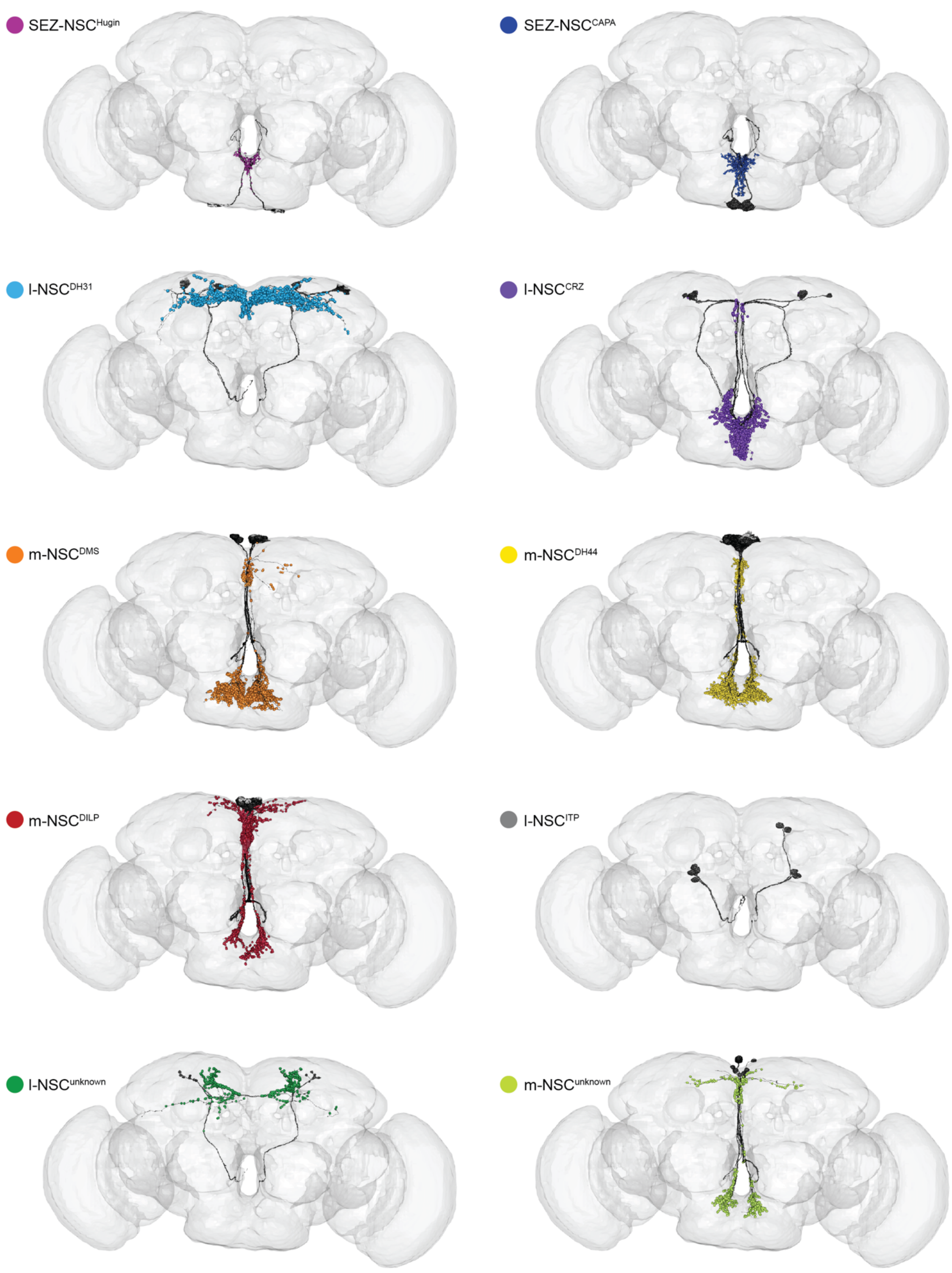

bioRxiv preprint doi: https://doi.org/10.1101/2024.08.28.609616; this version posted August 29, 2024. The copyright holder for this preprint (which was not certified by peer review) is the author/funder, who has granted bioRxiv a license to display the preprint in perpetuity. It is made available under aCC-BY 4.0 International license.

Figure 3 Supplement 1: Postsynaptic sites of NSC. Reconstructions of different NSC subtypes along with their postsynaptic sites. l-NSC^{ITP} are an exception and have very few postsynaptic sites.

bioRxiv preprint doi: https://doi.org/10.1101/2024.08.28.609616; this version posted August 29, 2024. The copyright holder for this preprint (which was not certified by peer review) is the author/funder, who has granted bioRxiv a license to display the preprint in perpetuity. It is made available under aCC-BY 4.0 International license.

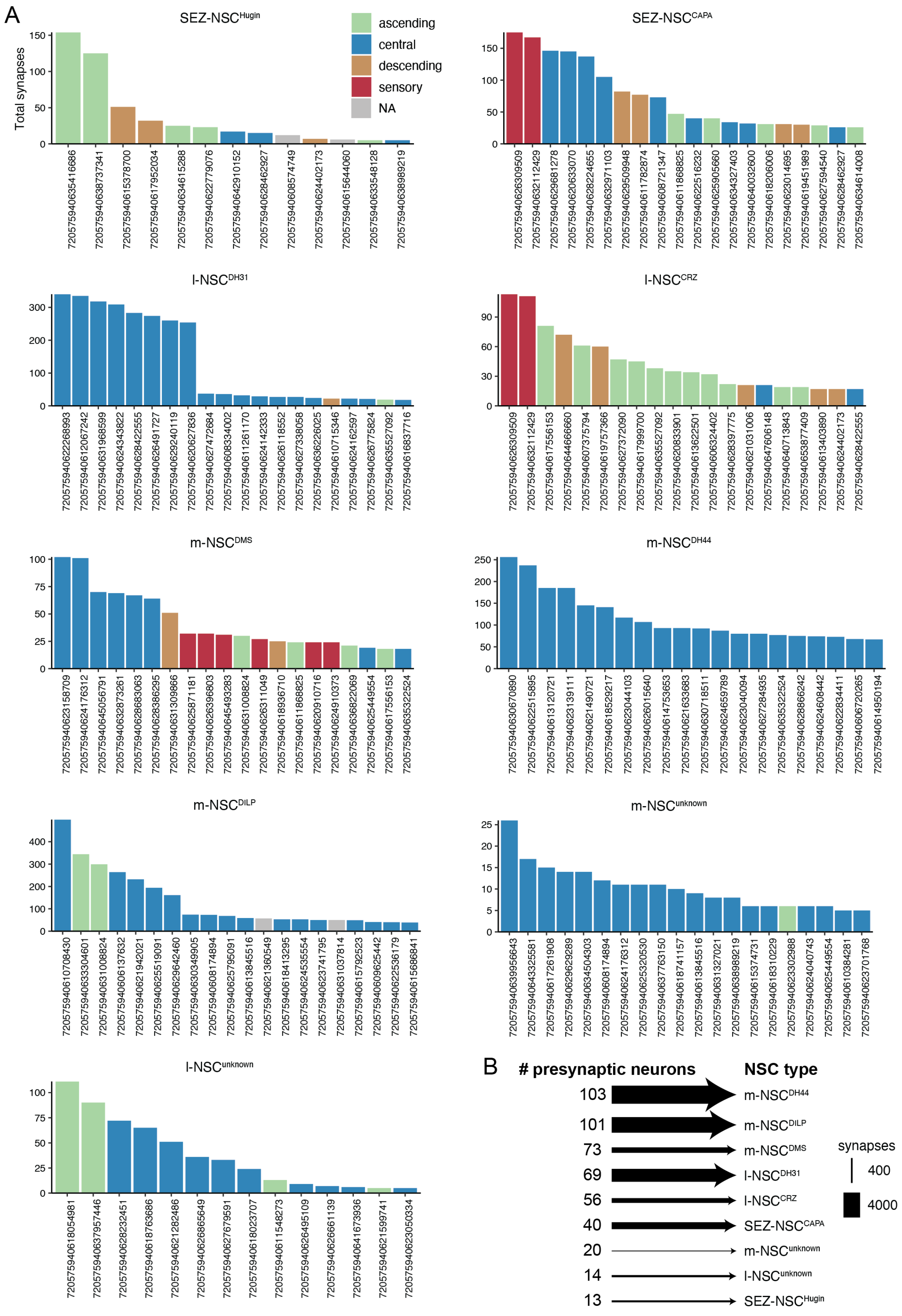

bioRxiv preprint doi: https://doi.org/10.1101/2024.08.28.609616; this version posted August 29, 2024. The copyright holder for this preprint (which was not certified by peer review) is the author/funder, who has granted bioRxiv a license to display the preprint in perpetuity. It is made available under aCC-BY 4.0 International license.

Figure 3 Supplement 2: Inputs to NSC subtypes. (A) Individual presynaptic partners of different NSC sorted based on the number of synapses. Presynaptic neurons are colored based on the super class they belong to. Only the top 20 neurons are shown. SEZ-NSC^{CAPA} and l-NSC^{CRZ} receive strong sensory inputs whereas l-NSC^{DH31}, m-NSC^{DH44} and m-NSC^{unknown} mostly receive inputs from central neurons. **(B)** Number of presynaptic neurons providing inputs to different types of NSC.

bioRxiv preprint doi: https://doi.org/10.1101/2024.08.28.609616; this version posted August 29, 2024. The copyright holder for this preprint (which was not certified by peer review) is the author/funder, who has granted bioRxiv a license to display the preprint in perpetuity. It is made available under aCC-BY 4.0 International license.

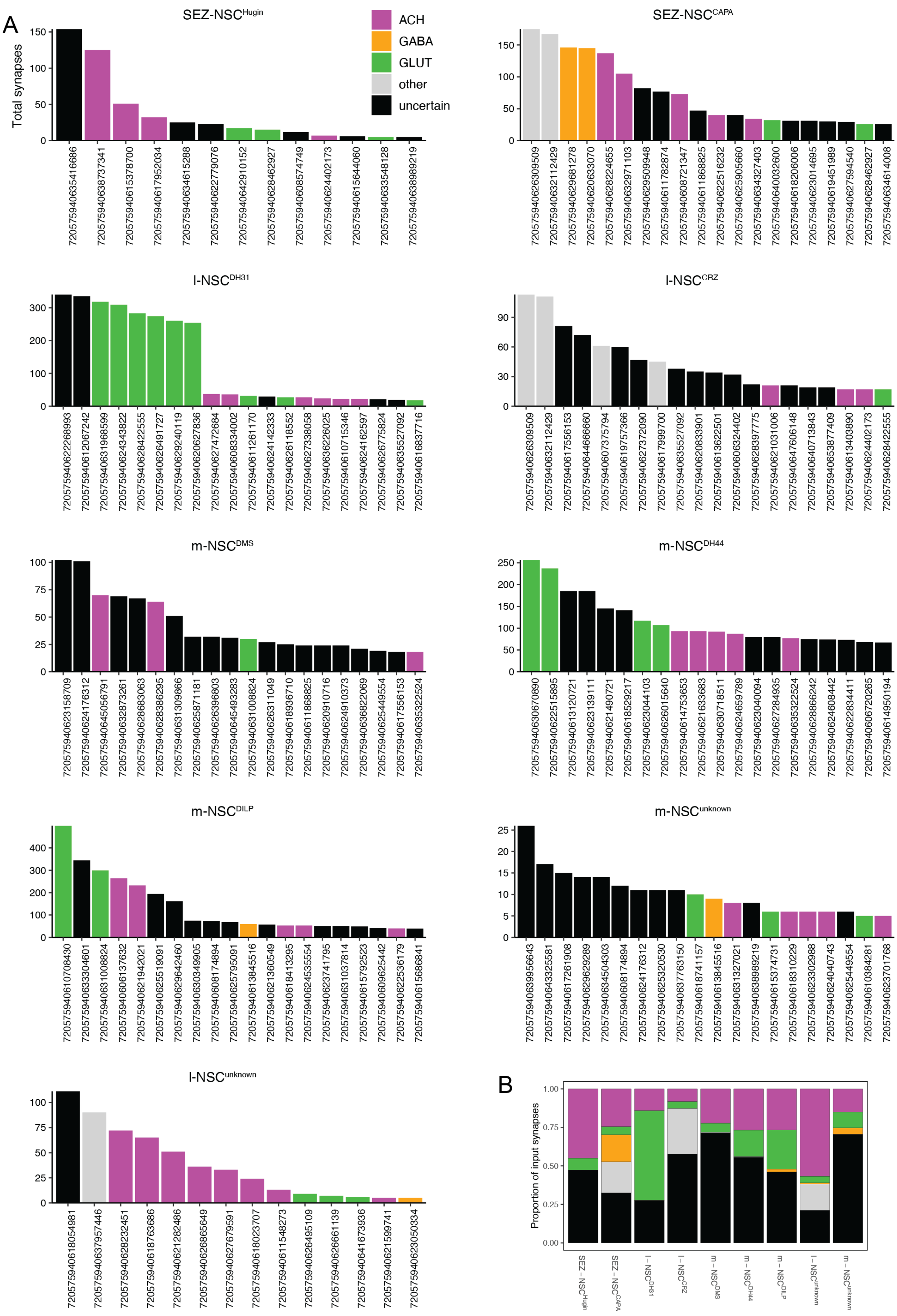

bioRxiv preprint doi: https://doi.org/10.1101/2024.08.28.609616; this version posted August 29, 2024. The copyright holder for this preprint (which was not certified by peer review) is the author/funder, who has granted bioRxiv a license to display the preprint in perpetuity. It is made available under aCC-BY 4.0 International license.

Figure 3 Supplement 3: Neurotransmitters providing inputs to NSC subtypes. (A) Individual presynaptic partners of different NSC sorted based on the number of synapses and colored based on their neurotransmitter identity. l-NSC^{DH31} and m-NSC^{DH44} receive strong glutamatergic inputs. **(B)** Input to NSC grouped by the neurotransmitters. Out of the three fast-acting neurotransmitters, GABA provides the least inputs.

bioRxiv preprint doi: https://doi.org/10.1101/2024.08.28.609616; this version posted August 29, 2024. The copyright holder for this preprint (which was not certified by peer review) is the author/funder, who has granted bioRxiv a license to display the preprint in perpetuity. It is made available under aCC-BY 4.0 International license.

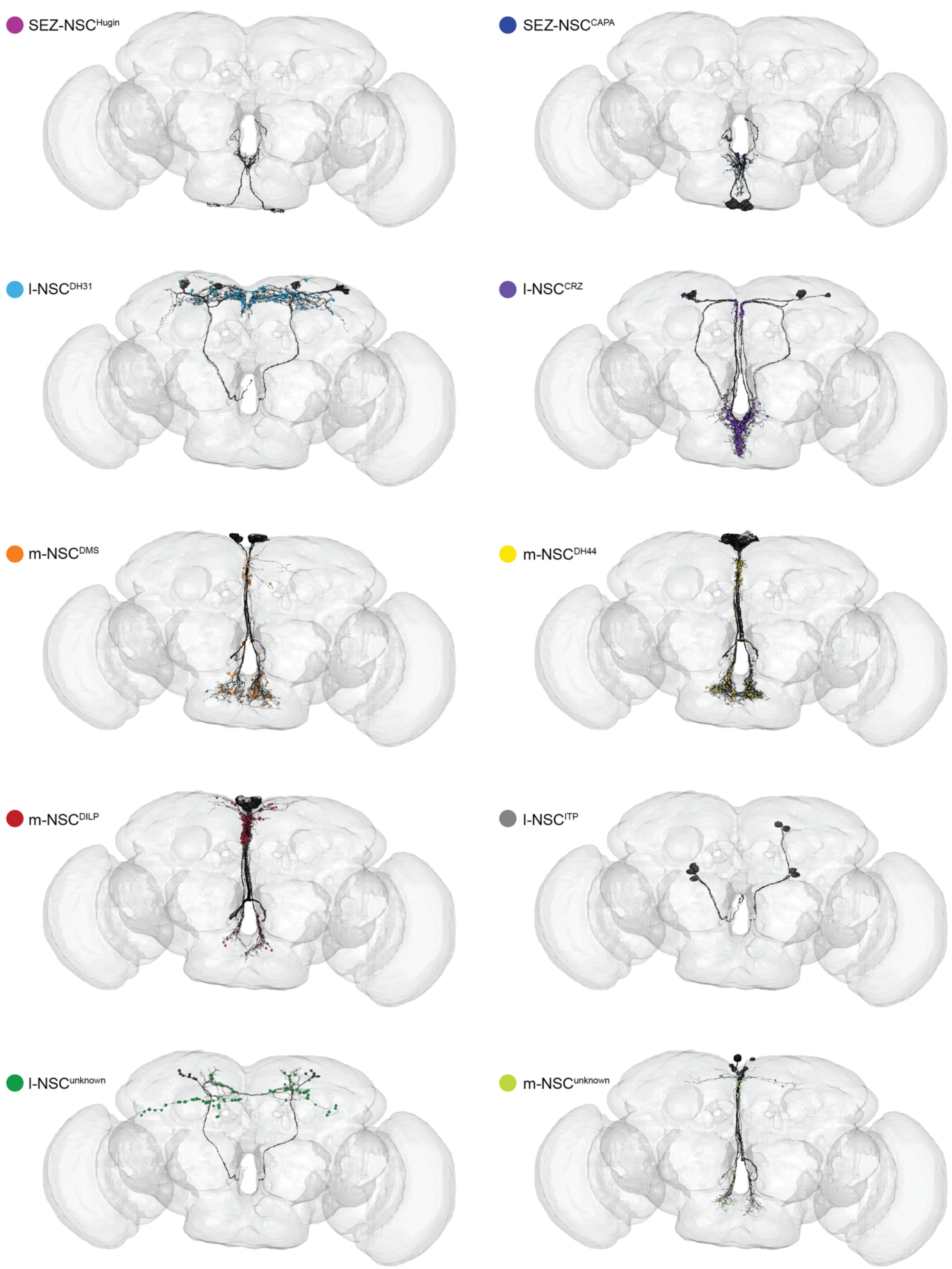

bioRxiv preprint doi: https://doi.org/10.1101/2024.08.28.609616; this version posted August 29, 2024. The copyright holder for this preprint (which was not certified by peer review) is the author/funder, who has granted bioRxiv a license to display the preprint in perpetuity. It is made available under aCC-BY 4.0 International license.

Figure 6 Supplement 1: Presynaptic sites of NSC. Reconstructions of different NSC subtypes along with their presynaptic sites. l-NSC^{CRZ} have several presynaptic sites in the SEZ.

bioRxiv preprint doi: https://doi.org/10.1101/2024.08.28.609616; this version posted August 29, 2024. The copyright holder for this preprint (which was not certified by peer review) is the author/funder, who has granted bioRxiv a license to display the preprint in perpetuity. It is made available under aCC-BY 4.0 International license.

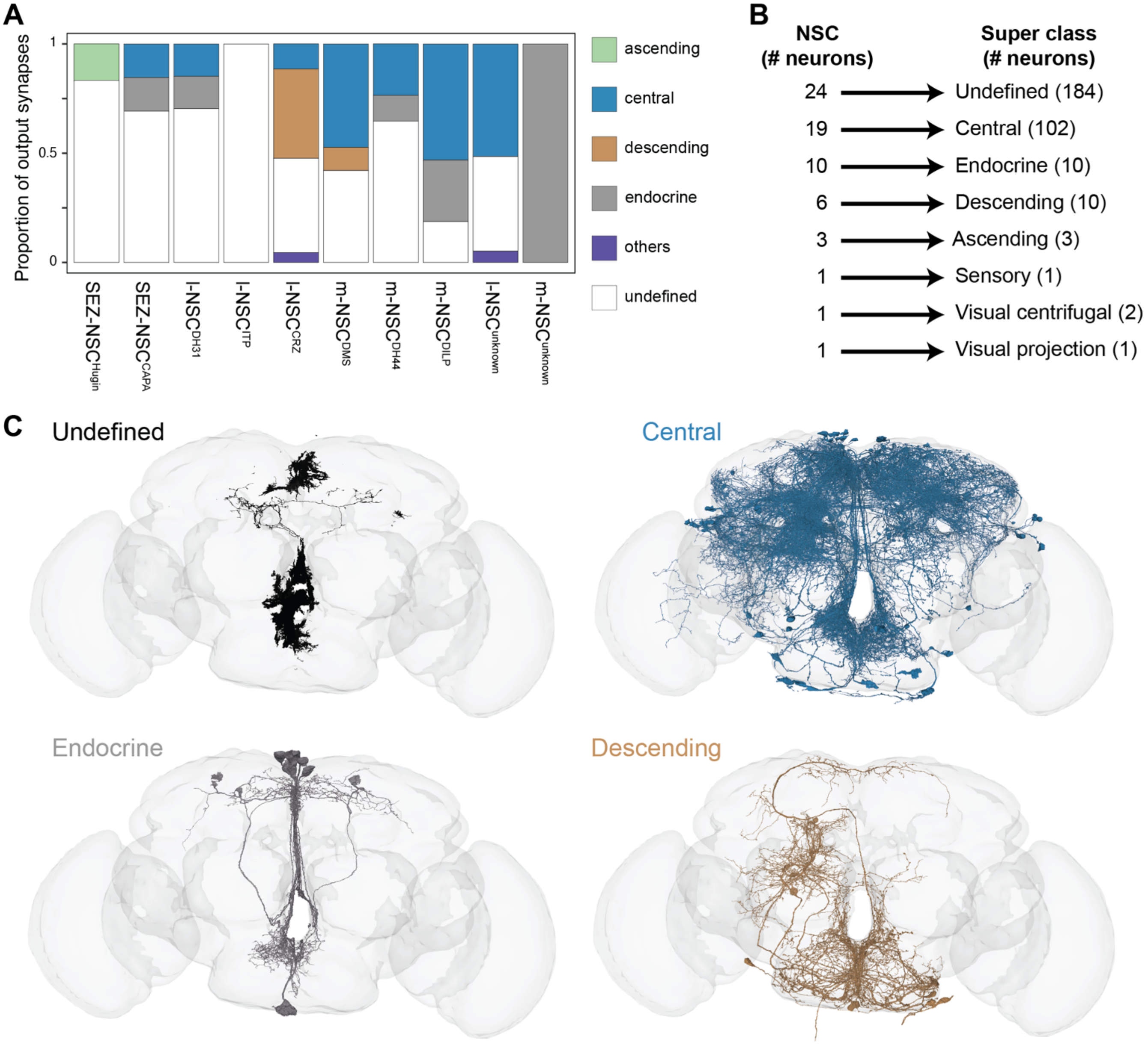

Figure 6 Supplement 2: Synaptic output from NSC based on a low synaptic threshold. **(A)** Proportion of outputs from different types of NSC to various neuronal super classes when the threshold for a significant connection is lowered to 2 synapses. **(B)** Output from NSC grouped by the neuronal super classes annotated in the FlyWire connectome. **(C)** Reconstructions of neurons receiving inputs from NSC. Cells belonging to top four super classes are shown. Note that most of the output from NSC is to partial fragments and non-neuronal cells (undefined), as well as central neurons.

bioRxiv preprint doi: https://doi.org/10.1101/2024.08.28.609616; this version posted August 29, 2024. The copyright holder for this preprint (which was not certified by peer review) is the author/funder, who has granted bioRxiv a license to display the preprint in perpetuity. It is made available under aCC-BY 4.0 International license.

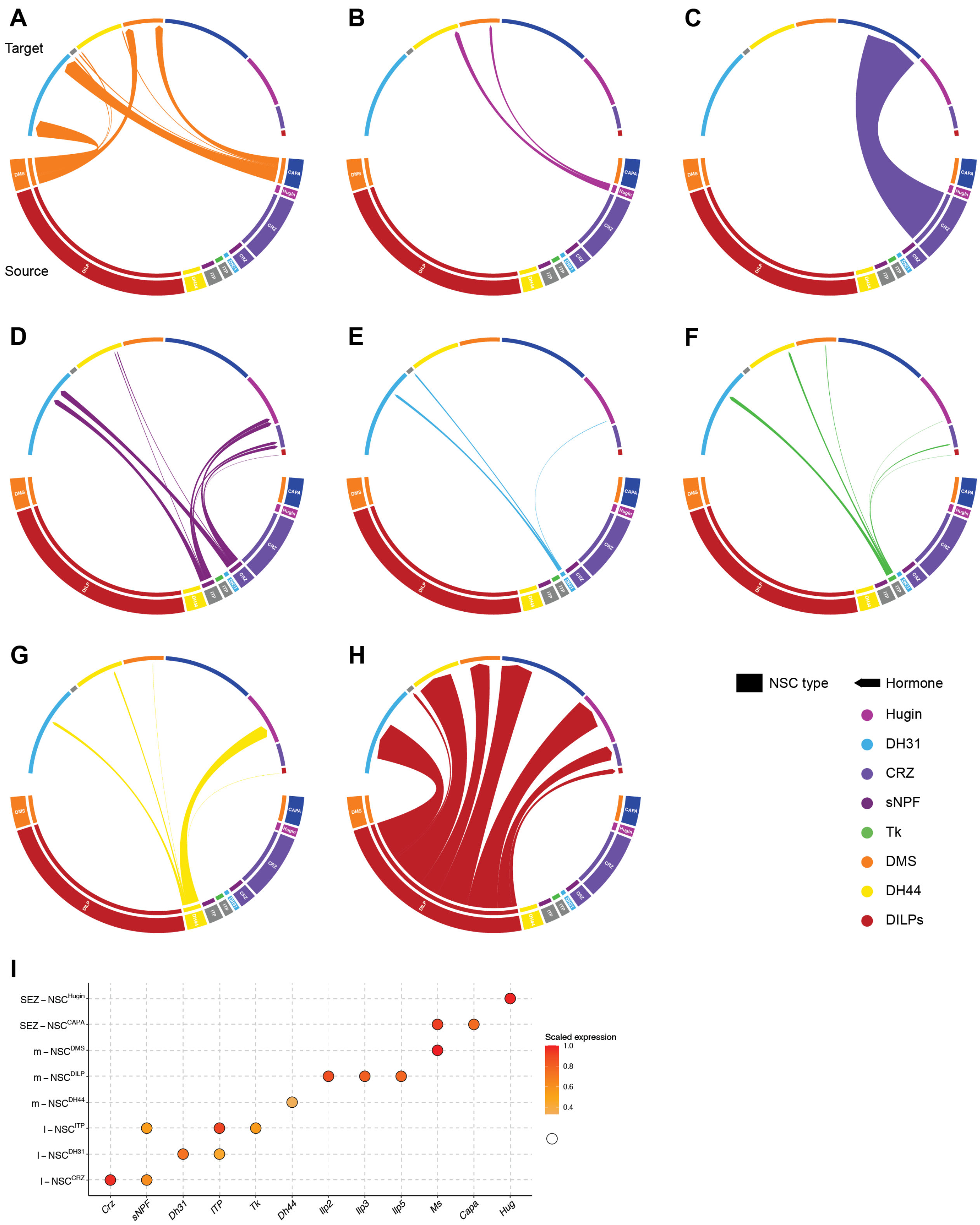

Figure 7 Supplement 1: Paracrine interconnectivity between NSC. NSCs subtypes targeted by **(A)** myosuppressin (DMS), **(B)** Hugin, **(C)** corazonin (CRZ), **(D)** short neuropeptide F (sNPF),

bioRxiv preprint doi: https://doi.org/10.1101/2024.08.28.609616; this version posted August 29, 2024. The copyright holder for this preprint (which was not certified by peer review) is the author/funder, who has granted bioRxiv a license to display the preprint in perpetuity. It is made available under aCC-BY 4.0 International license.

(E) diuretic hormone 31 (DH31), **(F)** tachykinin (TK), **(G)** diuretic hormone 44 (DH44) and **(H)** insulin-like peptides (DILPs). Ion transport peptide and CAPA pathways are not included because their receptors were not detected in these transcriptomes. **(I)** Dot plot showing the neuropeptides expressed in each NSC type following thresholding. The expression has been scaled and was used to generate the connectivity diagrams in Figure 7C and Figure 7 Supplement 1A-H.

bioRxiv preprint doi: https://doi.org/10.1101/2024.08.28.609616; this version posted August 29, 2024. The copyright holder for this preprint (which was not certified by peer review) is the author/funder, who has granted bioRxiv a license to display the preprint in perpetuity. It is made available under aCC-BY 4.0 International license.

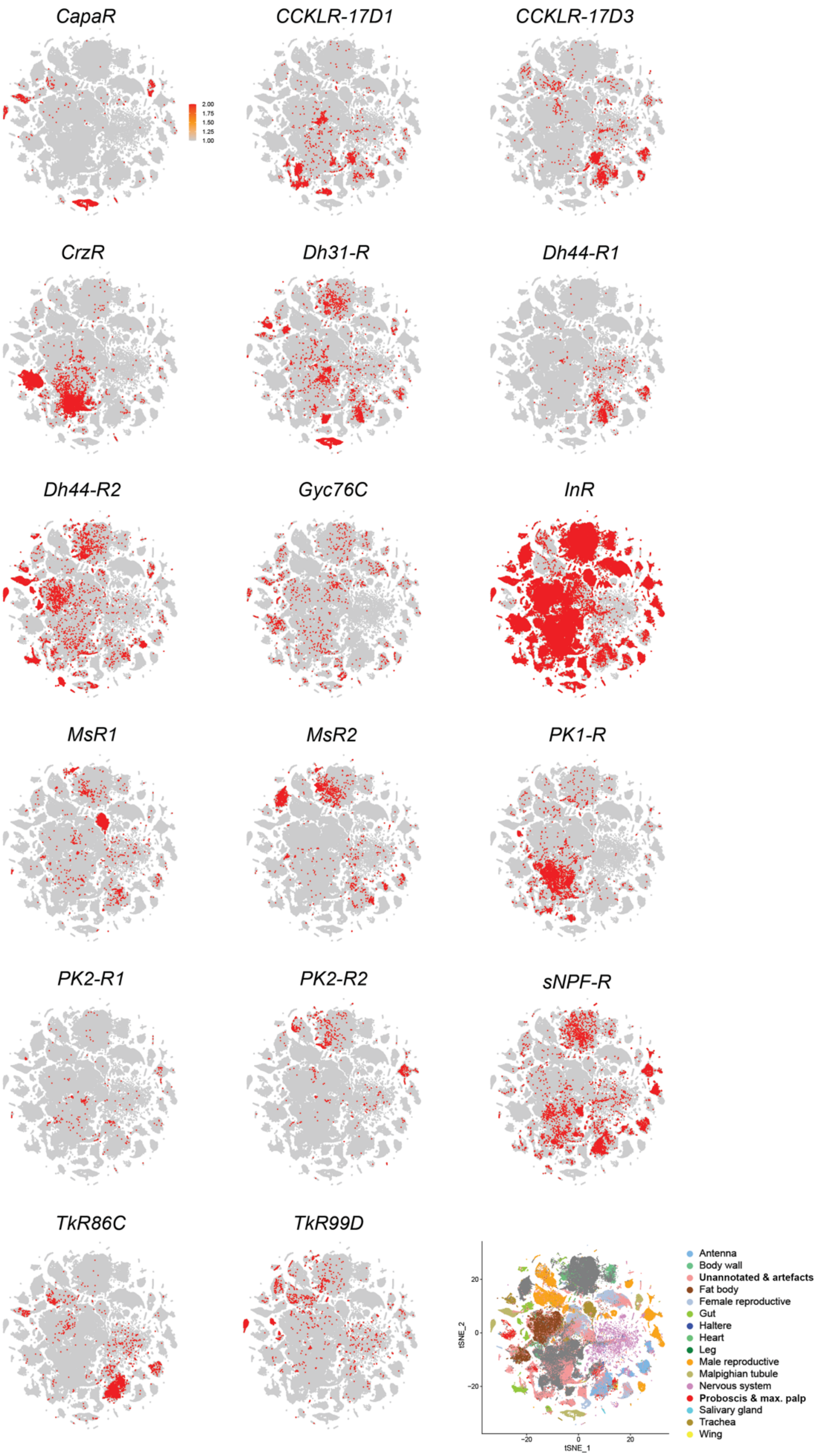

bioRxiv preprint doi: https://doi.org/10.1101/2024.08.28.609616; this version posted August 29, 2024. The copyright holder for this preprint (which was not certified by peer review) is the author/funder, who has granted bioRxiv a license to display the preprint in perpetuity. It is made available under aCC-BY 4.0 International license.

Figure 7 Supplement 2: Expression of receptors for hormones released from brain NSC. t-SNE plots showing expression of hormone receptors across single-cell transcriptomes from all *Drosophila* tissues (Li *et al.*, 2022). Note that some receptors such as *InR* and *sNPF-R* are broadly expressed whereas others such as *CapaR* and *PK2-R1* are sparsely expressed.

bioRxiv preprint doi: https://doi.org/10.1101/2024.08.28.609616; this version posted August 29, 2024. The copyright holder for this preprint (which was not certified by peer review) is the author/funder, who has granted bioRxiv a license to display the preprint in perpetuity. It is made available under aCC-BY 4.0 International license.

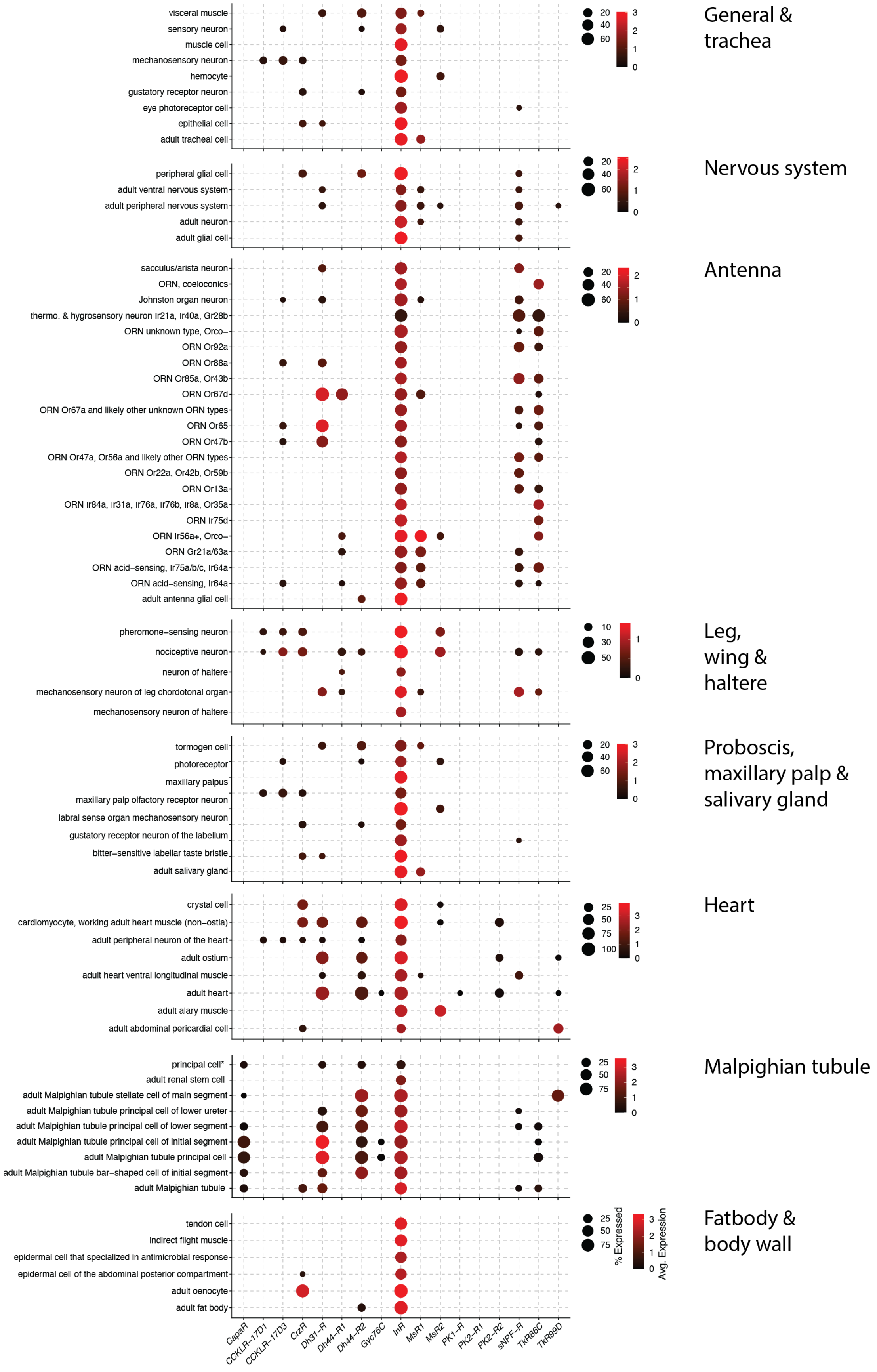

bioRxiv preprint doi: https://doi.org/10.1101/2024.08.28.609616; this version posted August 29, 2024. The copyright holder for this preprint (which was not certified by peer review) is the author/funder, who has granted bioRxiv a license to display the preprint in perpetuity. It is made available under aCC-BY 4.0 International license.

Figure 7 Supplement 3: Expression of hormone receptors in peripheral tissues. Dot plots showing expression of hormone receptors in different tissues at single-cell resolution. Expression of only those receptors whose corresponding neuropeptides are expressed in brain NSC are shown.

bioRxiv preprint doi: https://doi.org/10.1101/2024.08.28.609616; this version posted August 29, 2024. The copyright holder for this preprint (which was not certified by peer review) is the author/funder, who has granted bioRxiv a license to display the preprint in perpetuity. It is made available under aCC-BY 4.0 International license.

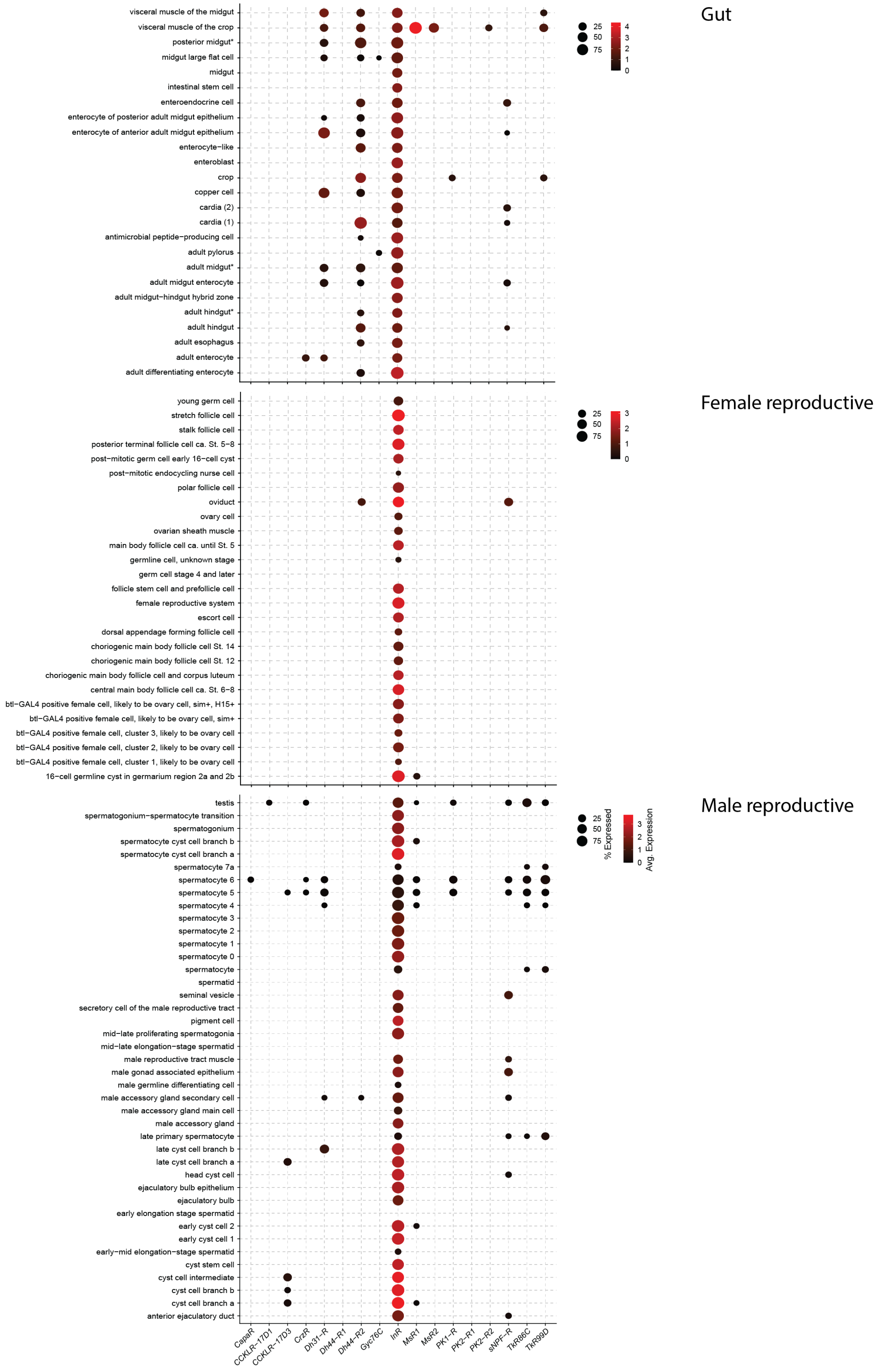

bioRxiv preprint doi: https://doi.org/10.1101/2024.08.28.609616; this version posted August 29, 2024. The copyright holder for this preprint (which was not certified by peer review) is the author/funder, who has granted bioRxiv a license to display the preprint in perpetuity. It is made available under aCC-BY 4.0 International license.

Figure 7 Supplement 4: Expression of hormone receptors in the gut and reproductive tissues. Dot plots showing expression of hormone receptors in the gut and reproductive tissues at single-cell resolution. Expression of only those receptors whose corresponding neuropeptides are expressed in brain NSC are shown.

bioRxiv preprint doi: https://doi.org/10.1101/2024.08.28.609616; this version posted August 29, 2024. The copyright holder for this preprint (which was not certified by peer review) is the author/funder, who has granted bioRxiv a license to display the preprint in perpetuity. It is made available under aCC-BY 4.0 International license.

Supplementary Tables

Supplementary Table 1. Fly strains used in this study.

Fly strain	BDRC Stock number / Reference
DH44-Gal4	51987
DILP2-Gal4	(Ikeya *et al.*, 2002)
DILP3-Gal4	
DILP5-Gal4	66008
MS-T2A-Gal4	84652
Gr64a-Gal4	57661
w^{1118}	5905
UAS-retro-Tango	(Sorkac *et al.*, 2023)
JFRC81-10xUAS-IVS-Syn21-GFP-p10 (EGFP)	(Pfeiffer *et al.*, 2012)
10xUAS-myr::GFP (myrGFP)	(Pfeiffer *et al.*, 2010)
UAS-NLS-mCherry	38425

Supplementary Table 2: Antibodies used for immunohistochemistry in this study.

Antibody	Immunogen	Dilution	Source / Reference
Primary antibodies			
chicken anti-GFP	Recombinant full-length GFP	1:1000	Abcam, RRID: AB_300798
guinea pig anti-RFP		1:5000	Gift from Dr. Susan Morton
mouse nc82 anti-Bruchpilot		1:50	(Wagh *et al.*, 2006)
rat anti-HA (3F10)	YPYDVPDYA	1:100	Roche
rabbit anti-CRZ	*Periplaneta americana* CRZ	1:1000	(Veenstra and Davis, 1993)
rabbit anti-DILP2	*Drosophila* DILP2	1:2000	(Veenstra *et al.*, 2008)
rabbit anti-DILP3	*Drosophila* DILP3	1:500	(Veenstra *et al.*, 2008)
rabbit anti-MS	*Locusta migratoria* MS	1:500	(Schoofs *et al.*, 1993)
rabbit anti-CAPA	*Rhodnius prolixus* CAPA-2	1:2000	Gift from Dr. Ian Orchard
Secondary antibodies			
goat anti-chicken Alexa Fluor® 488	IgY (H+L) chicken	1:1000	Thermo Fisher Scientific
donkey anti-guinea pig Alexa Fluor ® 555		1:1000	Thermo Fisher Scientific
goat anti-guinea pig Alexa Fluor® 647		1:1000	Thermo Fisher Scientific
donkey anti-rabbit Alexa Fluor ® 555		1:1000	Thermo Fisher Scientific
donkey anti-rat Alexa Fluor ® 555		1:1000	Thermo Fisher Scientific
donkey anti-rabbit Alexa Fluor ® 647		1:1000	Thermo Fisher Scientific
donkey anti-mouse Alexa Fluor ® 647		1:1000	Thermo Fisher Scientific

Supplementary Table 3: FlyWire cell IDs of identified NSC (see separate file)
